# The Antiperovskite‐Type Oxychalcogenides Ae_3_Q[GeOQ_3_] (Ae = Ba, Sr; Q = S, Se) with Large Second Harmonic Generation Responses and Wide Band Gaps

**DOI:** 10.1002/advs.202204755

**Published:** 2022-12-05

**Authors:** Shaoxin Cui, Hongping Wu, Zhanggui Hu, Jiyang Wang, Yicheng Wu, Hongwei Yu

**Affiliations:** ^1^ Tianjin Key Laboratory of Functional Crystal Materials Institute of Functional Crystal College of Materials Science and Engineering Tianjin University of Technology Tianjin 300384 P. R. China

**Keywords:** antiperovskite‐type, heteroanionic materials, NLO materials, oxychalcogenides, structure‐property relationship

## Abstract

Oxychalcogenides capable of exhibiting excellent balance among large second‐harmonic generation (SHG) response, wide band gap (*E*_g_), and suitable birefringence (Δ*n*) are ideal materials class for infrared nonlinear optical (IR NLO) crystals. However, rationally designing a new high‐performance oxychalcogenide IR NLO crystal still faces a huge challenge because it requires the optimal orientations of the heteroanionic groups in oxychalcogenide. Herein, a series of antiperovskite‐type oxychalcogenides, Ae_3_Q[GeOQ_3_] (Ae = Ba, Sr; Q = S, Se), which were synthesized by employing the antiperovskite‐type Ba_3_S[GeS_4_] as the structure template. Their structures feature novel three‐dimensinoal frameworks constructed by distorted [QAe_6_] octahedra, which are further filled by [GeOQ_3_] tetrahedra to form antiperovskite‐type structures. Based on the unique antiperovskite‐type structures, the favorable alignment of the polarizable [GeOQ_3_] tetrahedra and distorted [QAe_6_] octahedra have been achieved. These contribute the ideal combination of large SHG response (0.7–1.5 times that of AgGaS_2_), wide *E*_g_ (3.52–4.10 eV), and appropriate Δ*n* (0.017–0.035) in Ae_3_Q[GeOQ_3_]. Theoretical calculations and crystal structure analyses revealed that the strong SHG and wide *E*_g_ could be attributed to the polarizable [GeOQ_3_] tetrahedra and distorted [QAe_6_] octahedra. This research provides a new exemplification for the design of high‐performance IR NLO materials.

## Graphical abstract

The heteroanionic antiperovskite‐type infrared nonlinear optical materials, Ae_3_Q[GeOQ_3_] (Ae = Ba, Sr; Q = S, Se), have been synthesized. Their structures consist of the heteroanionic [GeOQ_3_] tetrahedra and the Q‐centered octahedral [QAe_6_] groups linked together through vertices to form the antiperovskite framework. They exhibit promising properties including high SHG responses, large *E*_g_ as well as appropriate birefringence.

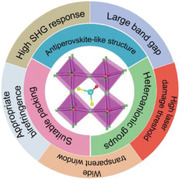

## 1 Introduction

Nonlinear optical (NLO) crystals are widely concerned due to their ability to expand the output spectral range of laser sources based on frequency conversion technology.^[^ ^1^ ^]^ During the past several decades, many excellent NLO crystals have been discovered, such as LiB_3_O_5_ (LBO),^[^ ^2^ ^]^ *β*‐BaB_2_O_4_ (BBO),^[^ ^3^ ^]^ KH_2_PO_4_ (KDP)^[^ ^4^ ^]^ and KTiOPO_4_ (KTP).^[^ ^5^ ^]^ However, in the infrared (IR) region, only AgGaS_2_ (AGS),^[^ ^6^ ^]^ AgGaSe_2_ (AGSe)^[^ ^7^ ^]^ and ZnGeP_2_ (ZGP)^[^ ^8^ ^]^ are commercialized and they contain some intrinsic drawbacks, such as low laser damage threshold (LDT) and two‐photon absorption (TPA), which prohibit their applications in high‐power conditions.^[^ ^9^ ^]^ Therefore, it is still urgent to explore new IR nonlinear optical (NLO) crystals with excellent performance.^[^ ^1^, ^10^ ^]^ For an IR NLO crystal, the crystallographically non‐centrosymmetric (NCS) structure is the prerequisite because only NCS structures can exhibit second‐order NLO properties. Beyond this, some harsh functional properties are necessary for the application of IR NLO crystals, including: i) high second‐harmonic generation (SHG) coefficient; ii) large band gap (*E*_g_); iii) appropriate birefringence (Δ*n*); iv) broad transmission window; and v) favorable physical and chemical stability and crystal growth habit.^[^ ^1c^ ^]^ Among them, the strong SHG response and wide *E*_g_ are generally conflicted. Therefore, it is a great challenge to reasonably design IR NLO crystal with excellent performance.

Some previous works have demonstrated that combining multiple anionic groups to construct a heteroanionic compound can provide a new avenue for materials to balance the above‐conflicted properties because the heteroanionic compounds can integrate the property superiority of different anion groups, for example, the strong SHG response and wide IR transmission of metal chalcogenides and the large *E*_g_ and high LDT of oxides.^[^ ^11^ ^]^ Therefore, the heteroanionic IR NLO crystals have become the current hotspots, and numerous high‐performance heteroanionic IR NLO crystals have been synthesized, which consist of the: chalcohalides, such as Ba_4_Ge_3_S_9_Cl_2_ (2.4 × AGS, 2.91 eV),^[^ ^12^ ^]^ [RbBa_2_Cl][Ga_4_S_8_] (1.0 × AGS, 3.30 eV),^[^ ^13^ ^]^ [Ba_4_Cl_2_][ZnGa_4_S_10_] (1.1 × AGS, 3.85 eV),^[^ ^14^ ^]^ Li[LiCs_2_Cl][Ga_3_S_6_] (0.7 × AGS, 4.18 eV);^[^ ^15^ ^]^ oxychalcogenides, such as BaGeOSe_2_ (1.1 × AGS, 3.2 eV),^[^ ^16^ ^]^ SrGeOSe_2_ (1.3 × AGS, 3.16 eV),^[^ ^17^ ^]^ Sr_2_ZnSn_2_OS_6_ (0.7 × AGS, 3.52 eV),^[^ ^18^ ^]^ Sr_2_GeGa_2_OS_6_ (1.7 × AGS, 3.15 eV),^[^ ^19^ ^]^ LaMGa_3_S_6_O (M = Ca; Sr) (0.9–1.0 × AGS, 3.21–3.27 eV);^[^ ^20^ ^]^ and oxyhalides, such as Pb_17_O_8_Cl_18_ (2 × AGS, 3.44 eV).^[^ ^21^ ^]^ Among them, although chalcohalides exhibit excellent properties, most of their birefringence is too small to achieve phase matching (PM). For oxyhalides, the cations containing lone pair electrons are the main contributors to the SHG response, but the selectivity of such ions is relatively limited. Whereas, the oxychalcogenides are particularly impressive because most of them can exhibit the optimal balance among the three critical parameters, SHG response, *E*_g_ and Δn. And the research for the oxychalcogenide NLO crystals is still in its infancy. Developing a rational structure designing strategy is quite essential for exploring new oxychalcogenide NLO crystals.

It is well known that materials’ properties are governed by their structures. In recent years, the perovskite structure with the general formula of ABX_3_ has been investigated extensively, especially in the fields of the photocatalytic and photovoltaic.^[^ ^22^ ^]^ Both A and B sites can be partially replaced by other ions with similar radius, and the crystal structure may be maintained. Antiperovskites have the same general formula as perovskite, but A and B are anions, and X is a cation, accordingly written as X_3_BA.^[^ ^23^ ^]^ Their properties can also be adjusted by the type of A and B sites. In addition, perovskites or antiperovskites usually have an ordered octahedral framework that facilitates the orientation alignment of A‐site groups. Hence, some excellent NLO crystals, such as K_3_B_6_O_10_Cl^[^ ^24^ ^]^ and K_3_B_6_O_10_Br,^[^ ^25^ ^]^ have been obtained in the field of NLO using a perovskite structure as template. However, antiperovskite templates have not been used in the field of IR NLO crystals. At the same time, we notice that chalcogenides condensed with the 14 group elements have exhibited a variety of structure units and featured abundant information on crystallographic chemistry, for example, [GeS_4_] tetrahedron, disulfide S_2_^2−^ and isolated S^2−^ anions. In the current research, we have realized that in S‐rich Ba_3_GeS_5_,^[^ ^26^ ^]^ its structure consists of the A‐site [GeS_4_] groups and the X_3_B‐sites S‐centered [SBa_6_] octahedra linked together through vertices to form the antiperovskite framework represented by X_3_BA (Figure S1, Supporting Information). In the antiperovskite‐type Ba_3_S[GeS_4_] structure, the A, B and X‐sites can be designed based on the well‐known rules for the antiperovskite types to optimize the functional properties of materials. The choice of the A‐site atoms/groups is limited by the Goldschmidt tolerance factor, $t=\left(r_{A}+r_{x}\right)/\sqrt{2}\left(r_{B}+r_{x}\right)$, where *r*_A_, *r*_B_ and *r*_x_ are the effective radii of A‐site, B‐site and X‐sites atoms/groups. When 0.85 < *t* < 1, the antiperovskite structure can form, but when it is smaller than 0.85, usually orthorhombic or lower crystallographic symmetry structures would form instead.^[^ ^23^ ^]^ The obtained value for Ba_3_S[GeS_4_] is 0.77, that is outside the boundary of the tolerance factor range, indicating unstability of the structure. Since the radius of the O^2−^ ions is much smaller than that of the S^2−^, the volume of the [GeO*_n_* Q*_m_*] (Q = S, Se) group is usually smaller than that of the [GeQ_4_] group, which opens up the possibility of the substitutions of the A‐site groups. Furthermore, because electronegativity, ionic radius, coordination capability and polarizability of oxygen anions are different from those of sulfur anions, which can induce larger distortion of metal‐centered coordination polyhedra to enlarge SHG response. With these ideas in mind, we have successfully designed and synthesized four new NCS and polar oxychalcogenides, Ae_3_Q[GeOQ_3_] (Ae = Ba, Sr; Q = S, Se), which possess antiperovskite‐type structures. In Ae_3_Q[GeOQ_3_] (Ae = Ba, Sr; Q = S, Se), the individual [QAe_6_] Q‐centered octahedra create a distorted antiperovskite framework, which regulates the arrangement of highly distorted [GeOQ_3_] tetrahedra to produce high SHG responses (0.7–1.5 × AGS). In addition, Ae_3_Q[GeOQ_3_] (Ae = Ba, Sr; Q = S, Se) exhibit large *E*_g_ and appropriate Δ*n* indicating that they are promising IR NLO crystals. Herein, their syntheses, performance characterization, theoretical calculations as well as related comparisons are systemically investigated.

## 2 Results and Discussion

Ae_3_Q[GeOQ_3_] (Ae = Ba, Sr; Q = S, Se) were synthesized by the solid‐state reaction in sealed silica tubes. Their purities were checked by powder X‐ray diffraction (PXRD) (Figure S2, Supporting Information). Energy dispersive spectroscopy (EDS) analyses of Ae_3_Q[GeOQ_3_] (Ae = Ba, Sr; Q = S, Se) give the Ae/Ge/Q atomic ratio of 34.30–36.28%:12.62–15.22%:50.47–52.40%, which is approximately equal to the theoretical one, 37.50%:12.50%:50.00% (Figure S3, Supporting Information). The single crystal diffraction results demonstrate that Ae_3_Q[GeOQ_3_] (Ae = Ba, Sr; Q = S, Se) are isostructural and crystallize in the NCS and polar orthorhombic space group, *Pca*2_1_ (No. 29, Table S1, Supporting Information). Here, only Ba_3_S[GeOS_3_] is selected to display the crystal structure. The asymmetric unit of Ba_3_S[GeOS_3_] contains three Ba atoms, one Ge atom, one O atom and four S atoms. Each Ge atom is coordinated with one O atom and three S atoms forming the distorted [GeOS_3_] tetrahedra with the short Ge—O distance of 1.755(7) Å and long Ge—S distances in the scope of 2.207(3)–2.223(3) Å, respectively. There are three Ba coordination environments: O‐sharing Ba(1)OS_7_, Ba(2)OS_6_, and Ba(3)OS_6_ polyhedra. In these polyhedra, Ba—O distances are in the range of 2.616(8)–2.706(8) Å, and Ba—S distances are in the scope of 3.057(3)–3.529(3) Å (Figure S4 and Table S2, Supporting Information). For Ba_3_Se[GeOSe_3_] and Sr_3_Q[GeOQ_3_] (Q = S, Se), they have similar coordination with Ba_3_S[GeOS_3_]. The chosen bond distances and angles have been listed in Table S2, Supporting Information. The bond valence sums calculation values on Ba/Sr, Ge, O and S/Se atoms are 1.91–2.13, 3.97–4.11, 1.88–2.06 and 1.91–2.20 (Table S3, Supporting Information), respectively.

**Figure 1:**
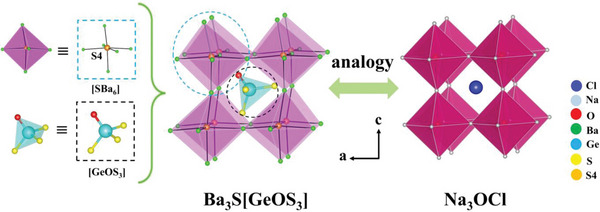
The analogy between the antiperovskite‐type structure of Ba_3_S[GeOS_3_] and the antiperovskite structure of Na_3_OCl.

The structure of Ba_3_S[GeOS_3_] is shown in **Figure** **1**. It contains the isolated S(4)^2−^ anions which are not coordinated with Ge atom and only surrounded by six Ba ions to form a [S(4)Ba_6_] octahedra (Figure 1). The adjacent four [S(4)Ba_6_] octahedra connected by corner‐sharing form an irregularity void, where the [GeOS_3_] group is located (Figure 1). By analogy with the antiperovskite Na_3_OCl (Figure 1),^[^ ^27^ ^]^ the positions of Cl^−^ anions are occupied by the [GeOS_3_] groups, the positions of O^2−^ anions are similar to those of S(4)^2−^ anions and the position of Na^+^ cations are similar to the positions of Ba^2+^ cations. Hence, the title compounds can be represented as Ae_3_Q[GeOQ_3_] (Ae = Ba, Sr; Q = S, Se).

**Figure 2:**
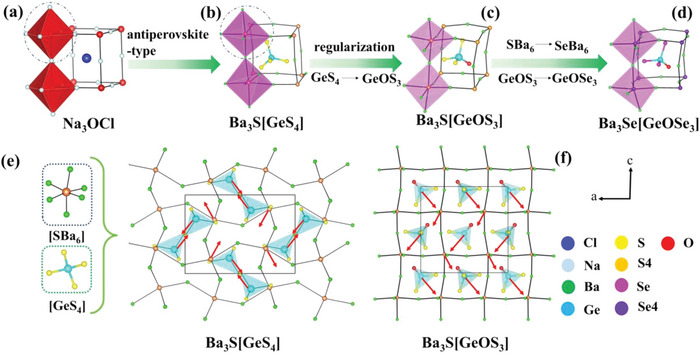
a) The antiperovskite structure of Na_3_OCl; b) the antiperovskite‐type structure of Ba_3_S[GeS_4_]; c) Ba_3_S[GeOS_3_] and d) Ba_3_Se[GeOSe_3_]; e) view of the three‐dimensional (3D) net structure of Ba_3_S[GeS_4_] formed by isolated [GeS_4_] tetrahedra and [SBa_6_] octahedra along *b*‐axis; f) view of the 3D net structure of Ba_3_S[GeOS_3_] formed by isolated [GeOS_3_] tetrahedra and [SBa_6_] octahedra along *b*‐axis. The red arrows indicate the direction of the polarity.

Furthermore, the structure of Ae_3_Q[GeOQ_3_] (Ae = Ba, Sr; Q = S, Se) can be viewed to evolve from Ba_3_S[GeS_4_] (**Figure** **2**a–d). It is worth noting that Ba_3_S[GeS_4_] crystallizes in a centrosymmetric (CS) space group *Pnma* (No. 62), while Ae_3_Q[GeOQ_3_] (Ae = Ba, Sr; Q = S, Se) crystallizes in the NCS and polar orthorhombic space group *Pca*2_1_ (No. 29). In perovskite structure, the size of the A‐site group dictates the distortion of the perovskite lattice.^[^ ^28^ ^]^ Antiperovskite has similar structure rules to perovskite, so the A‐site groups’ substitution is responsible for their structural transformation from CS to NCS. For Ba_3_S[GeS_4_], the Ge—S bond distances in the relatively regular [GeS_4_] tetrahedra have only a small change from 2.1733(12) to 2.2194(17) Å. More importantly, the antiparallel arrangement of these [GeS_4_] tetrahedra causes their local dipole moments to cancel (Figure 2e). Moreover, although the [S(4)Ba_6_] octahedron has a distortion (Figure 2e) with the S—Ba distances ranging from 3.1326(17) to 3.4479(17) Å, these [S(4)Ba_6_] octahedra have an opposite arrangement (Figure 2e). These make Ba_3_S[GeS_4_] crystallize in a CS structure. However conversely, in Ba_3_S[GeOS_3_], for the [GeOS_3_] tetrahedra, the Ge—O distances (1.755(7) Å) are obviously shorter than that of Ge—S (2.207(3)‐2.223(10) Å), and the relatively wide bond angle ranges are also obtained in S—Ge—S and S—Ge—O bonds (from 105.85(14) to 114.6(3)°). Those indicate that the [GeOS_3_] tetrahedra have a strong distortion. In addition, the alignment orientation of both [GeOS_3_] and [S(4)Ba_6_] are along the *c*‐axis (Figure 2f), which results in the polar structure of Ba_3_S[GeOS_3_]. The same situation is also observed in Ba_3_Se [GeOSe_3_], Sr_3_S[GeOS_3_], and Sr_3_Se[GeOSe_3_]. Remarkably, compared with S‐based compounds, the Se‐based ones have more distorted tetrahedra, meaning that the Se‐based compounds have greater polarity than S‐based ones. In brief, the A‐site groups’ substitution has achieved the NCS and polar structure of Ae_3_Q[GeOQ_3_] (Ae = Ba, Sr; Q = S, Se).

The tolerance factor is a widely used parameter to evaluate the stability of perovskite, and it is also applicable to ionic antiperovskite.^[^ ^23^ ^]^ Therefore, we calculate the tolerance factor of four target compounds and parent structure. The distortion parameters such as the distortion index (D) and bond angle variance (*σ* ^2^) are given by Equations (1) and (2) implemented in the Vesta software, where *l*_i_ and *l*_av_ are the individual and average Q—Ae bond length, respectively, and *r* is the effective ionic radius of the ion.^[^ ^22^ ^]^

(1) $$D=\frac{1}{6}\sum_{i}^{6}\frac{\left|l_{i}-l_{\mathrm{av}}\right|}{l_{\mathrm{av}}}$$

(2) $$\sigma^{2}=\sum_{i=1}^{12}{\left(\theta_{i}-90\right)}^{2}/11$$

**Figure 3:**
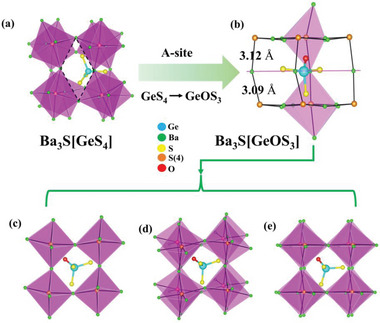
a) The structure of Ba_3_S[GeS_4_]; b) view of Ba_3_S[GeOS_3_] along [$\bar{1}\bar{1}0$]; c) the structure of Ba_3_S[GeOS_3_] along the *b*‐axis; d) Ba_3_S[GeOS_3_] structure along the *c*‐axis; e) Ba_3_S[GeOS_3_] structure along the a‐axis.

The rational substitution from Ba_3_S[GeS_4_] to Ba_3_S[GeOS_3_] can be interpreted as follows. As shown in Table S4, Supporting Information, [SBa_6_] octahedra in Ba_3_S[GeS_4_] have large distortion, while its structure tolerance factor is much less than 0.85, but it still maintains the antiperovskite configuration, indicating that unstability of the structure. Meanwhile, we also calculated the global instability index (GII), which is an important parameter for evaluating structural stability. The global instability index (GII) value of Ba_3_S[GeS_4_] is 0.41 vu greater than 0.20 vu (Table S5, Supporting Information), suggesting that its structure is unstable under tension. Structurally, in Ba_3_S[GeS_4_], the [SBa_6_] octahedra are inclined to connect an approximate rhomboid channel, and its A‐site group [GeS_4_] isn't in the center of the void. As shown in **Figure** **3**a, it can be seen that the A‐site group deviates significantly from the center of the cavity, leaving the structure in a metastable state. For Ba_3_S[GeOS_3_], the volume of [GeOS_3_] tetrahedron is smaller than that of [GeS_4_] tetrahedron (Table S4, Supporting Information), which means that the [GeOS_3_] tetrahedra fit better into void space among the [SBa_6_] octahedra. The [SBa_6_] octahedra are less inclined and form an approximate square channel, and the [GeOS_3_] tetrahedron is located in the center of the void. The Ge atom of [GeOS_3_] tetrahedron lies in the center of the plane formed by four Ba atoms at the vertex of the octahedra, and three S atoms point to the center of the plane composed of S(4) atoms, respectively, while the O atom points to the plane center composed of three Ba atoms at the vertex of an octahedron (Figure 3b–e). Obviously, such a structure is more stable than the parent structure Ba_3_S[GeS_4_]. Thus, the substitution of the A‐site group achieves the structure transformation from CS to NCS, and also increased structural stability.

**Figure 4:**
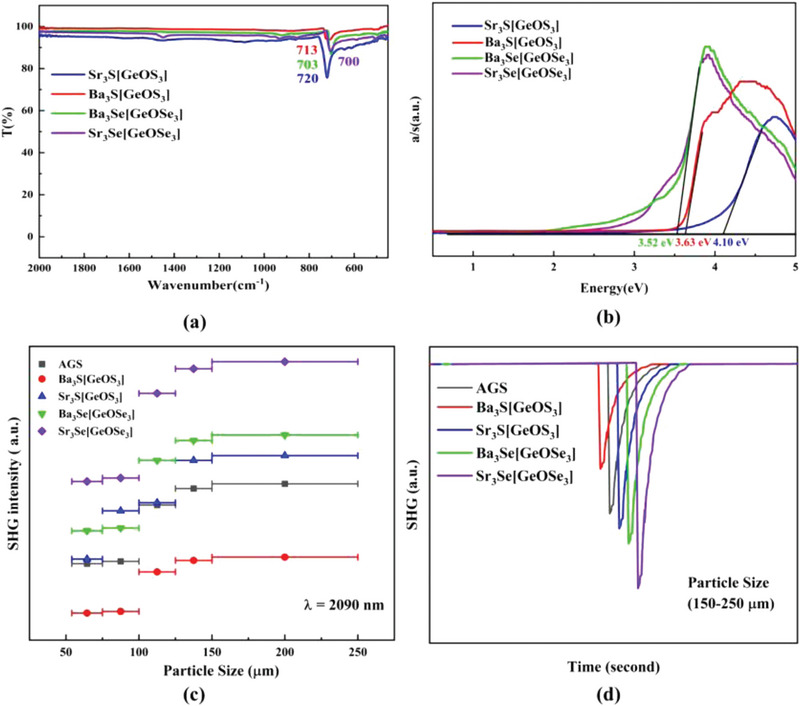
a) The IR spectra of title compounds; b) optical bandgaps of title compounds; c) SHG intensities versus particle sizes for Ae_3_Q[GeOQ_3_] (Ae = Ba, Sr; Q = S, Se) and AGS as a reference; d) SHG intensities of Ae_3_Q[GeOQ_3_] (Ae = Ba, Sr; Q = S, Se) and AGS at particle size of 150–250 µm.

The optical spectra (employing IR spectroscopies and measuring the *E*_g_ values) of four compounds were measured (**Figure** **4**a,b and Figure S5, Supporting Information). Clearly, there is only one absorption peak (≈700 cm^−1^) observed in their respective IR spectra, which can be attributed to the Ge—O stretching mode. Remarkably, compared with the absorption peaks (720 and 713 cm^−1^, respectively) of S‐based Sr_3_S[GeOS_3_] and Ba_3_S[GeOS_3_], the absorption peaks of Se‐based Sr_3_Se[GeOSe_3_] and Ba_3_Se[GeOSe_3_] (700 and 703 cm^−1^, respectively) move to the longer wavelengths (Figure 4a). That indicates the heavier Se atoms are helpful for further widening the IR transparent region of materials. In addition, among the four compounds, Sr_3_S[GeOS_3_] has the strongest absorption, while Ba_3_S[GeOS_3_] has the weakest absorption. Clearly, the order of their IR absorption strengths is also consistent with the magnitude order of dipole moments of Ge—O bonds (Table S6, Supporting Information). These indicate the small Ge—O dipole moment might be favorable for weakening the IR absorption of materials. More importantly, for all compounds, they have no obvious absorption before 700 cm^−1^ (≈14 µm), indicating these materials can cover the two critical atmospheric windows (3–5 and 8–12 µm), which are important for applications such as telecommunications, laser guidance and explosives detection.

Moreover, the optical *E*_g_ of Ae_3_Q[GeOQ_3_] (Ae = Ba, Sr; Q = S, Se) are shown in Figure 4b, which are converted to the absorption according to the following Kubelka–Munk Equation (3):^[^ ^29^ ^]^

(3) $$F\;\left(R\right)=\frac{K}{S}=\frac{{\left(1-R\right)}^{2}}{2R}$$

where *R* is the reflectance, *K* is the absorption, and *S* is the scattering. On their absorption curves, elongating the linear part to zero, wide band gaps of 3.52, 3.52, 3.63 and 4.10 eV can be obtained for Ba_3_Se[GeOSe_3_], Sr_3_Se[GeOSe_3_], Ba_3_S[GeOS_3_] and Sr_3_S[GeOS_3_], respectively. Clearly, for the Ge‐containing oxides, their *E*_g_ are generally around 5.0 eV, such as BaGeO_3_ (4.96 eV),^[^ ^30^ ^]^ Li_3_Rb_3_Ge_6_O_15_ (5.63 eV).^[^ ^31^ ^]^ While the *E*_g_ of chalcogenides are usually around or smaller than 3.0 eV, such as Ba_3_GeS_5_ (3.0 eV),^[^ ^26^ ^]^ Mg_2_GeSe_4_ (2.02 eV)^[^ ^32^ ^]^ and Mg_2_SnS_4_ (2.05 eV).^[^ ^33^ ^]^ For Ae_3_Q[GeOQ_3_] (Ae = Ba, Sr; Q = S, Se), their *E*_g_ are located in the region between those of oxides and chalcogenides. These are reasonable and indicate that introducing the O^2−^ anions in chalcogenides can indeed effectively improve the band gaps of chalcogenides. In addition, it should also be noticed that the smallest *E*_g_ among Ae_3_Q[GeOQ_3_] (Ae = Ba, Sr; Q = S, Se) is 3.52 eV (Sr_3_Se[GeOSe_3_] and Ba_3_Se[GeOSe_3_]), which is still larger than those of commercial IR NLO crystals AGS (2.70 eV)^[^ ^6^ ^]^ and ZGP (2.0 eV),^[^ ^8^ ^]^ implying that they may exhibit higher LDTs.

The LDTs of Ae_3_Q[GeOQ_3_] (Ae = Ba, Sr; Q = S, Se) have been measured based on the single‐pulse power LDT method with AGS as the reference. The results show that the powder LDTs are around 86.53 MW cm^−2^ for both Sr_3_S[GeOS_3_] and Ba_3_S[GeOS_3_], and 59.35 and 32.79 MW cm^−2^ for Sr_3_Se[GeOSe_3_] and Ba_3_Se[GeOSe_3_], respectively. They are around 16 ×, 11 ×, and 6 × AGS (5.4 MW cm^−2^) under the same condition, respectively.^[^ ^1b^ ^]^ Such high LDTs of Ae_3_Q[GeOQ_3_] (Ae = Ba, Sr; Q = S, Se) will favor their potential application in high‐powder laser fields.

To evaluate the NLO coefficients, the powder SHG responses of Ae_3_Q[GeOQ_3_] (Ae = Ba, Sr; Q = S, Se) and AGS were explored on the basis of Kurtz–Perry technique (a 2.09 µm laser as the fundamental light).^[^ ^34^ ^]^ The curve of the SHG signal versus particle size is showed in Figure 4c, their SHG intensities increase with the particle size, indicating the PM property of the compounds. As shown in Figure 4d, they exhibit SHG responses around 0.7, 1.1, 1.2, and 1.5 times of that of AGS at the largest particle sizes of 150−250 µm, respectively. They are comparable with other oxychalcogenide NLO materials such as SrZn_2_S_2_O (2 × KDP),^[^ ^35^ ^]^ Sr_5_Ga_8_O_3_S_14_ (0.8 × AGS),^[^ ^36^ ^]^ SrGeOS_2_ (0.4 × AGS),^[^ ^37^ ^]^ BaGeOS_2_ (0.5 × AGS),^[^ ^37^ ^]^ Sr_3_Ge_2_O_4_Se_3_ (0.8 × AGS)^[^ ^38^ ^]^ and Sr_3_[SnOSe_3_][CO_3_] (1.0 × AGS) (Table S7, Supporting Information).^[^ ^39^ ^]^

According to the structure–property relationship, the SHG responses of materials mainly originate from the superposition of the microscopic NLO polarizability of asymmetric building units.^[^ ^40^ ^]^ As the basic microscopic NLO primitives, the [GeOQ_3_] tetrahedra show high distortion due to the large difference in the bond distances of Ge—Q and Ge—O. And the contribution of Q(4)^2−^ anions to SHG response can't be ignored. Therefore, to better understand the contribution of the [GeOQ_3_] tetrahedra and [QAe_6_] octahedra to the large NLO effect, an analysis of out‐of‐center distortions of [QAe_6_] octahedra and the dipole moments of the polar groups in Ae_3_Q[GeOQ_3_] were carried out.

**Figure 5:**
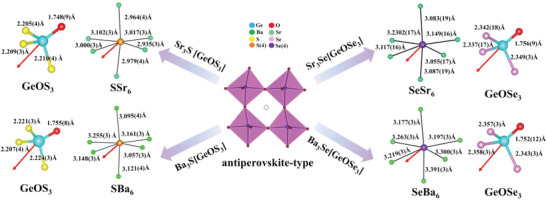
[GeOQ_3_] and [QAe_6_] polyhedra in Ae_3_Q[GeOQ_3_] (Ae = Ba, Sr; Q = S, Se). The red arrows indicate the approximate direction of their dipole moments.

All [QAe_6_] octahedra in Ae_3_Q[GeOQ_3_] (Ae = Ba, Sr; Q = S, Se) as shown in **Figure** **5**, and the levels of out‐of‐center distortions can be calculated using the equation proposed by Halasyamani.^[^ ^41^ ^]^ Octahedra distortion, Δ*d* parameter, is defined as below:

(4) $$\begin{array}{ccc} \Delta d & = & \frac{\left|\left(M-O1\right)-\left(M-O4\right)\right|}{\left|\mathrm{COS}\;\theta_{1}\right|}+\frac{\left|\left(M-O2\right)-\left(M-O5\right)\right|}{\left|\mathrm{COS}\;\theta_{2}\right|}\\ & & +\;\frac{\left|\left(M-O3\right)-\left(M-O6\right)\right|}{\left|\mathrm{COS}\;\theta_{3}\right|} \end{array}$$

where the pairs (O1, O4), (O2, O5) and (O3, O6) are the alkali‐earth metal atoms that make up the octahedron, which are located in opposite positions to each other. The Δ*d* values are 0.21, 0.22, 0.25 and 0.29 for Sr_3_S[GeOS_3_], Sr_3_Se[GeOSe_3_], Ba_3_S[GeOS_3_] and Ba_3_Se[GeOSe_3_], respectively. These values all fall into the second category defined by Halasyamani, with a magnitude located in the range of Δ*d* = 0.05–0.4; following the criteria proposed by the author, these figures correspond to a weak distortion. Table S6, Supporting Information, summarizes the magnitudes of the [QAe_6_] octahedra distortions. Remarkably, the Δ*d* of [QAe_6_] octahedra in Ae_3_Q[GeOQ_3_] are all smaller than that of Ba_3_S[GeS_4_] (0.45). Therefore, Ae_3_Q[GeOQ_3_] is closer to the undistorted antiperovskite structure than Ba_3_S[GeS_4_]. This implies that Ae_3_Q[GeOQ_3_] have a more regularly arranged octahedra framework which facilitates the orderly packing of [GeOQ_3_] tetrahedra, leading to large SHG responses.

In the Ae_3_Q[GeOQ_3_] (Ae = Ba, Sr; Q = S, Se), the calculated dipole moments of the Ge—Q and Ge—O are 23.06–33.98 D and 13.32–13.59 D, respectively. The net dipole moments of 9.75–20.58 D were obtained by a vector sum of the dipole moments of [GeOS_3_] polyhedra (Table S6, Supporting Information). It is worth noting that in two sulfides, the magnitude of the dipole moment of [GeOS_3_] (9.75–11.76 D) and [S(4)Ae_6_] (8.22–9.39 D) groups are comparable, while in both selenides, the magnitude of the dipole moment of [Se(4)Ae_6_] (7.69–8.78 D) is about half that of [GeOSe_3_] (17.91–20.58 D) (Table S6, Supporting Information). These confirm that both the [GeOQ_3_] and [QAe_6_] groups have an important contribution for the large SHG responses of Ae_3_Q[GeOQ_3_]. In addition, in order to compare with other oxychalcogenides more conveniently, we normalized the dipole moment (Table S8, Supporting Information). The normalized dipole moment of [GeOSe_3_] in Sr_3_Se[GeOSe_3_] (0.0225 D/Å^3^) is larger than these of [GeOSe_3_] in Sr_3_Ge_2_O_4_Se_3_ (0.0132 D/Å^3^), [GeO_2_Se_2_] in BaGeOSe_2_ (0.0217 D/Å^3^). The normalized dipole moment of [GeOS_3_] in Sr_3_S[GeOS_3_] (0.0142 D/Å^3^) is larger than these of [GeO_2_S_2_] in BaGeOS_2_ (0.0104 D/Å^3^), [GeO_2_S_2_] in SrGeOS_2_ (0.0065 D/Å^3^). These analysis results of the normalized dipole moment also agree with the experimental SHG response intensities of these compounds.

**Figure 6:**
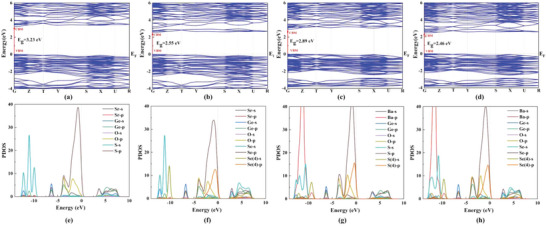
Band structures and density of states (DOS) diagrams of (a,e) Sr_3_S[GeOS_3_], (b,f) Sr_3_Se[GeOSe_3_], (c,g) Ba_3_S[GeOS_3_], and (d,h) Ba_3_Se[GeOSe_3_].

In order to further study the structure–property relationship, theoretical calculations based on density functional theory (DFT) methods were carried out (**Figure** **6** and Figures S6–S9, Supporting Information). The calculated band structures based on the Perdew–Burke–Ernzerhof (PBE) functional (Figure 6a–d) show the direct *E*_g_ of 3.23, 2.55, 2.89 and 2.46 eV for Sr_3_S[GeOS_3_], Sr_3_Se[GeOSe_3_], Ba_3_S[GeOS_3_] and Ba_3_Se[GeOSe_3_], respectively. Because the DFT method generally underestimates the value of *E*_g_, the smaller experimental values are observed. Figure 6e–h shows the partial density of states (PDOS) of Ae_3_Q[GeOQ_3_], they exhibit the similar PDOS. For Sr_3_S[GeOS_3_] and Ba_3_S[GeOS_3_], the valence band maximum (VBM) is mostly from the O 2*p*, S 3*p* orbitals with a little Sr 5*s*/Ba 6*s* orbital and the conduction band minimum (CBM) is mostly dominated by the Ge 4*s*, S 3*p* orbitals, with a little Ge 4*p*, O 2*p* orbitals. For Sr_3_Se[GeOSe_3_] and Ba_3_Se[GeOSe_3_], their VBMs are mostly from the O 2*p*, Se 4*p* orbitals, with a little Se 4*s*, Sr 5*s*/Ba 6*s* orbitals, and their CBMs are mainly dominated by the Se 4*p*, Ge 4*s* and Se 4*s* orbitals, with a little O 2*p* orbital. More specifically, there are two different types of S/Se ions in the title compounds, that is, the S/Se(1, 2, 3) ions in [GeOQ_3_] tetrahedra and S/Se(4) ions in [QAe_6_] octahedra. In order to better show the contributions of different S/Se ions on band structures,^[^ ^42^ ^]^ the 3*s*/4*s* and 3*p*/4*p* orbitals of S/Se(4) ions are also drawn in their DOS individually, and Figure S6, Supporting Information, shows the plots of the charge densities of the VBM and the CBM of title compounds. It is clear that the 3*p*/4*p* orbitals of S/Se(4) ions have almost equal contributions to their VBMs with the S/Se ions in the [GeOQ_3_] tetrahedra. Therefore, the optical *E*_g_ of Ae_3_Q[GeOQ_3_] (Ae = Ba, Sr; Q = S, Se) are predominantly determined by the electron transitions from both S/Se ions of octahedral center and [GeOQ_3_] tetrahedra to Ge^4+^ cations. That is, the optical properties of Ae_3_Q[GeOQ_3_] (Ae = Ba, Sr; Q = S, Se) originate from the distorted [QAe_6_] octahedra and [GeOQ_3_] tetrahedra.

In addition, we also noticed that the PBE usually underestimates the *E*_g_, while the Heyd–Scuseria–Ernzerhof (HSE06) hybrid functional can provide more accurate *E*_g_ values. So, we also calculated the *E*_g_ of Sr_3_S[GeOS_3_] with the HSE06 hybrid functional. It shows the *E*_g_ of Sr_3_S[GeOS_3_] is 4.26 eV (Figure S7, Supporting Information), which is indeed closer to the experimental value (4.10 eV). Besides, we also find that the same conclusion could be obtained from both calculations based on PBE and HSE06, that is, the [SSr_6_] octahedra and [GeOS_3_] groups have the main contribution for the VBM and the CBM of Sr_3_S[GeOS_3_] (Figure 6 and Figure S7, Supporting Information). But the calculations with the HSE06 hybrid functional are very time‐consuming and four compounds are iso‐structural. So, for the other compounds, only the calculated results from PBE functional were discussed.

Furthermore, the Δ*n* are important for determining the PM ability of materials. Hence, the Δ*n* of Ae_3_Q[GeOQ_3_] (Ae = Ba, Sr; Q = S, Se) were calculated based on their electronic structures. As shown in Figure S8, Supporting Information, the Δ*n* values of Ae_3_Q[GeOQ_3_] were calculated at 2.09 µm, in which the static Δ*n* values of Ae_3_Q[GeOQ_3_] (Ae = Ba, Sr; Q = S, Se) were 0.017, 0.021, 0.030 and 0.035, respectively. These values are consistent with the generally accepted ones of moderate Δ*n* and will be favorable for materials to achieve the PM. In order to show the relationship between birefringence and PM, we also selected Sr_3_S[GeOS_3_] and Sr_3_Se[GeOSe_3_] as representatives and calculated their PM wavelength ranges based on the calculated birefringence (Figure S10, Supporting Information). The results show that these birefringence values are sufficient to support the PM of Ae_3_Q[GeOQ_3_] (Ae = Ba, Sr; Q = S, Se).

**Figure 7:**
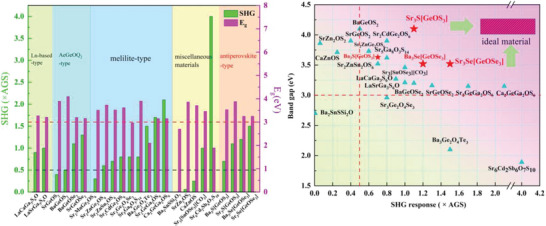
Classification and performance comparison of known IR NLO oxychalcogenides.

In addition, we noticed that the oxychalcogenide IR NLO crystals can well balance the conflicted relationship between large SHG response and wide *E*_g_. They have been extensively studied and these reported oxychalcogenides so far can consist of the Ln‐based type,^[^ ^20^ ^]^ AeGeOQ_2_‐type (Ae = Ba, Sr; Q = S, Se),^[^ ^16^, ^17^, ^37^ ^]^ melilite‐type^[^ ^18^, ^19^, ^36^, ^38^, ^43^ ^]^ and miscellaneous materials,^[^ ^34^, ^39^, ^44^ ^]^ as shown in **Figure** **7**. But remarkably, the title compounds represent a new type of antiperovskite‐type oxychalcogenides. Compared with other reported oxychalcogenides materials, the antiperovskite‐type Sr_3_Se[GeOSe_3_], Ba_3_Se[GeOSe_3_] and Sr_3_S[GeOS_3_] can exhibit excellent comprehensive performance, including strong SHG responses, large *E*_g_ and PM capability. These indicate that antiperovskite‐type oxychalcogenide would be a bright material category for exploring new IR NLO materials.

## 3 Conclusion

In summary, a series of NCS and polar antiperovskite‐type oxychalcogenides Ae_3_Q[GeOQ_3_] (Ae = Ba, Sr; Q = S, Se) have been successfully designed and obtained by high‐temperature solid‐state reactions. In their structures, highly distorted [GeOQ_3_] groups were introduced to change the lattice distortion of the antiperovskite‐type structure and reduced the distortion of [QAe_6_] octahedra to form the ordered 3D framework, which is conducive to the uniform alignment orientation of [GeOQ_3_] tetrahedra. Thereby, Ae_3_Q[GeOQ_3_] feature highly oriented [GeOQ_3_] tetrahedra and [QAe_6_] octahedra connected by corner‐sharing, which make major contributions to their SHG effects. Ae_3_Q[GeOQ_3_] all exhibit strong phase‐matching SHG responses of 0.7–1.5 × AGS, appropriate birefringence, and large *E*_g_. These indicate that regulating the orientation of basic building units through the antiperovskite‐type template will be a feasible method for designing new IR oxychalcogenide NLO materials with very comprehensive properties.

## 4 Experimental Section

### Reagent

All staring materials, Ba (99.99%), Ge (99.99%), Se (99.999%), S (99.999%), SrS (99.99%), SrSe (99.99%), GeSe_2_ (99.99%) and GeO_2_ (99.9%) were directly purchased from Aladdin Co., Ltd. without further purification. The binary materials BaSe and BaS were synthesized by heating a mixture of stoichiometric elements in vacuum flame sealed silicon tubes. All manipulations were carried out in an Ar‐filled glove box.

### Single Crystal Synthesis

Single crystals of Sr_3_S[GeOS_3_] and Ba_3_S[GeOS_3_] were synthesized using 3:0.5:0.5:1 ratios of SrS/BaS, GeO_2_, Ge, and S/Se with a total mass of 500 mg as the starting materials. After being ground to fine powder, the mixtures were packed into quartz tubes and further evacuated to 1 × 10^−3^ Pa, and then sealed by flame. The tubes were put into a muffle furnace, heated from room temperature to 950 °C within 6 h and kept at this temperature for 1 day, gradually cooled to 700 °C at a rate of 5 °C h^−1^, and finally the furnace was turned off. The single crystals of Sr_3_S[GeOS_3_] and Ba_3_S[GeOS_3_] were obtained.

The synthesis process of Sr_3_Se[GeOSe_3_] and Ba_3_Se[GeOSe_3_] were similar to that of Sr_3_S[GeOS_3_], except for the maximum temperature (900 °C). The different synthesized temperatures were mainly because of their different thermal stabilities. As shown in Figure S11, Supporting Information, for S‐based compounds, their main phase of Sr_3_S[GeOS_3_] and Ba_3_S[GeOS_3_] could be synthesized in the range of 900–950 °C, and they would decompose when the temperature was higher than 950 °C. While for Se‐based compounds, their pure phase could be obtained at 850–900 °C, and the samples would decompose when the temperature was higher than 900 °C.

### Structure Determination

PXRD analysis was performed in the angle range of 2*θ* = 10–70°, with a scan step width of 0.01° and a step time of 2 s using automated SmartLab 3KW powder X‐ray diffractometer with a diffracted monochromator set for Cu‐K_*α*_ (*λ* = 1.54057 Å) radiation. The purity of compounds Ae_3_Q[GeOQ_3_] (Ae = Ba, Sr; Q = S, Se) was verified by PXRD. The crystal structures of Ae_3_Q[GeOQ_3_] (Ae = Ba, Sr; Q = S, Se) were measured by single‐crystal XRD on a Bruker SMART APEX III CCD diffractometer using Mo‐K_*α*_ radiation (*λ* = 0.71073 Å) at 273–296 K and the data were integrated with the SAINT program.^[^ ^45^ ^]^ All calculations were implemented with programs from the SHELXTL crystallographic software package.^[^ ^46^ ^]^ Their crystal structures were solved by direct methods using SHELXS and refined with full‐matrix least‐squares methods on F^2^ with anisotropic thermal parameters for all atoms.^[^ ^47^ ^]^ The crystallographic data of the structures reported in this article are stored at the Cambridge Crystallographic Data Center with the CCDC No. 2202130–2202133. The data can be obtained free of charge from the Cambridge Crystallography Data Center at www.ccdc.cam.ac.uk/data_request/cif. Table S1, Supporting Information, demonstrated the crystal data and structure refinement parameters. Tables S2 and S3, Supporting Information, listed some structural parameters containing interatomic distances and angles, final refined atomic positions, and isotropic thermal parameters, respectively.

### Energy‐Dispersive Spectroscopy

Microprobe elemental analyses and the elemental distribution maps were measured on a field‐emission scanning electron microscope (Quanta FEG 250) made by FEI.

### Spectral Measurement

Optical diffuse reflectance spectra of Ae_3_Q[GeOQ_3_] (Ae = Ba, Sr; Q = S, Se) were measured on Shimadzu SolidSpec‐3700DUV with BaSO_4_ as a reference. The band gaps were estimated on basis of the absorption spectrum that was derived from the reflection spectrum using the Kubelka–Munk formula.^[^ ^29^ ^]^ The IR spectra were recorded on a Fourier transform IR spectrometer using Nicolet iS50FT.

### SHG Measurement

Optical SHG responses of Ae_3_Q[GeOQ_3_] (Ae = Ba, Sr; Q = S, Se) and benchmark AGS were evaluated by the modified Kurtz–Perry method,^[^ ^34^ ^]^ and a 2090 nm laser was used as incident radiation. Polycrystalline powders Ae_3_Q[GeOQ_3_] (Ae = Ba, Sr; Q = S, Se) and AGS were sieved into several distinct particle size ranges (54–75, 75–100, 100–125, 125–150, and 150–250 µm) to accomplish the PM measurements (Figure S12, Supporting Information). The SHG signals were detected by a charge‐coupled device. The AGS samples with similar particle size ranges were selected as the references.

### LDT Measurements

The LDTs of the Ae_3_Q[GeOQ_3_] (Ae = Ba, Sr; Q = S, Se) were measured using a high‐power laser irradiation of 1064 nm (pulse width *τ*_p_ = 10 ns) by the single‐pulse method and AGS served as a reference.^[^ ^48^ ^]^ The measurement processes were performed by gradually increasing the laser power until the damaged spot was observed.

### Computational Methods

The band structures, the PDOS of Ae_3_Q[GeOQ_3_] (Ae = Ba, Sr; Q = S, Se) were calculated by the CASTEP package based on DFT.^[^ ^49^ ^]^ The pseudopotential was set as norm‐conserving pseudopotential (NCP) and the exchange–correlation energy chosen was the generalized gradient approximation (GGA) parametrized by Perdew–Burke–Ernzerhof (PBE) functional.^[^ ^50^ ^]^ The orbital electrons of Sr: 4s^2^4p^6^5s^2^, Ba: 5s^2^5p^6^6s^2^, Ge: 4s^2^4p^2^, O: 2s^2^2p^4^, S: 3s^2^3p^4^, and Se 4s^2^4p^4^ were treated as valence electrons. The plane wave energy cutoff energy was set at 810.0 eV with a grid of Monkhorst–Pack k‐point meshes of 3 × 5 × 3.^[^ ^51^ ^]^ Since the PBE usually underestimates the *E*_g_, the Heyd–Scuseria–Ernzerhof (HSE06) hybrid functional was chosen to provide more accurate *E*_g_ values and the related parameter settings are the same as for PBE. For calculating the optical properties, the same Monkhorst‐pack grid and plane‐wave cutoff energy were used and the dielectric function is defined as *ε*(*ω*) = *ε*_1_(*ω*) + *iε*_2_(*ω*),^[^ ^52^ ^]^ in which real part *ε*_1_(*ω*), refractive index *n*(*ω*) were obtained by the Kramers–Kronig transform.^[^ ^53^ ^]^ The optical property calculations were scissor corrected (0.87, 0.97, 0.74 and 1.06 eV for Sr_3_S[GeOS_3_], Sr_3_Se[GeOSe_3_], Ba_3_S[GeOS_3_] and Ba_3_Se[GeOSe_3_], respectively) by the energy gap difference between the PBE and the experimental values.

### Statistical Analyses

The statistical results of band gaps and SHG responses of all oxychalcogenides IR NLO materials in Figure 7 and Table S7, Supporting Information, were applied on basis of the published paper.

## Conflict of Interest

The authors declare no conflict of interest.

## Supporting information

Supporting Information

## Data Availability

The data that support the findings of this study are available from the corresponding author upon reasonable request.
