# Supplementary material for: The Antiperovskite‐Type Oxychalcogenides Ae3Q[GeOQ3] (Ae = Ba, Sr; Q = S, Se) with Large Second Harmonic Generation Responses and Wide Band Gaps
Source: Adv Sci (Weinh). 2022 Dec 5;10(4):2204755. doi: 10.1002/advs.202204755 (PMC9896038; doi:10.1002/advs.202204755)
Supplement: Supplementary file 1 — Supporting Information [file ADVS-10-2204755-s001.pdf]

Supporting Information

The Antiperovskite-type Oxychalcogenides $Ae_3Q[GeOQ_3]$ (Ae = Ba, Sr; Q = S, Se) with Large Second Harmonic Generation Responses and Wide Band Gaps

*Shaoxin Cui, Hongping Wu, Zhanggui Hu, Jiyang Wang, Yicheng Wu and Hongwei Yu**

Table S1. Crystallographic data and refinement details for title compounds.

Empirical formula	$Sr_3S[GeOS_3]$	$Sr_3Se[GeOSe_3]$	$Ba_3S[GeOS_3]$	$Ba_3Se[GeOSe_3]$
Formula weight	479.69	667.34	628.85	816.4
Temperature (K)	293(2)	295(2)	273(2)	293(2)
Crystal system	Orthorhombic	Orthorhombic	Orthorhombic	Orthorhombic
Space group	*Pca*2(1)	*Pca*2(1)	*Pca*2(1)	*Pca*2(1)
Z	4	4	4	4
a (Å)	11.8253(19)	12.2366(13)	12.3522(7)	12.761(2)
b (Å)	5.9824(9)	6.2152(6)	6.2585(4)	6.4937(9)
c (Å)	11.6632(19)	12.0263(12)	12.1740(8)	12.5058(19)
D_c (g cm^{-3})	3.862	4.846	4.438	5.233
μ (mm^{-1})	23.823	36.497	16.359	28.101
F(000)	872	1160	1088	1376
Radiation	Mo-K_α (λ = 0.71073)	Mo-K_α (λ = 0.71073)	Mo-K_α (λ = 0.71073)	Mo-K_α (λ = 0.71073)
2θ range(°)	3.405 to 27.507	3.278 to 27.525	3.255 to 27.509	3.137 to 27.542
Reflections collected	7645	10784	10353	12339
Indep. Reflns/ Rint	1886/0.0568	2041/0.0609	2147/0.0501	2381/0.0664
GOOF on F^2	1.098	1.111	1.035	1.076
R_1[a], wR_2 (I >2σ(I))[b]	0.0355, 0.0686	0.0298, 0.0529	0.0275, 0.0508	0.0332, 0.0653
R_1, wR_2 (all data)	0.0447, 0.0741	0.0375, 0.0568	0.0339, 0.0533	0.0401, 0.0680
largest diff. peak and hole (e·Å^{-3})	0.971, -1.499	1.107, -1.369	1.033, -1.573	1.341, -1.701

[a] $R_1 = \Sigma||F_o| — |F_c||/\Sigma|F_o|$, [b] $wR_2 = \Sigma w(F_o^2 — F_c^2)^2/\Sigma w(F_o^2)^2]^{1/2}$

Table S2. Selected bond distances (Å) and angles (degrees) of title compounds

	$Sr_3S[GeOS_3]$		
Ge(1)-O(1)	1.747(8)	S(3)-Sr(1)#2	3.159(3)
Ge(1)-S(1)	2.205(3)	S(3)-Sr(1)#1	3.169(3)
Ge(1)-S(2)	2.209(3)	S(3)-Sr(2)#3	3.169(3)
Ge(1)-S(3)	2.210(4)	S(3)-Sr(3)	3.554(3)
S(1)-Sr(1)#4	3.006(4)	O(1)-Sr(2)	2.501(8)
S(1)-Sr(3)#4	3.060(3)	O(1)-Sr(1)	2.553(8)
S(1)-Sr(1)#1	3.175(4)	O(1)-Sr(3)	2.567(8)
S(1)-Sr(3)#5	3.223(3)	S(4)-Sr(2)	2.935(3)
S(2)-Sr(2)#5	3.041(4)	S(4)-Sr(3)#7	2.964(4)
S(2)-Sr(3)#5	3.132(3)	S(4)-Sr(3)#4	2.980(4)
S(2)-Sr(2)#1	3.162(4)	S(4)-Sr(1)#4	2.999(3)
S(2)-Sr(3)#6	3.184(3)	S(4)-Sr(1)#8	3.018(3)
S(3)-Sr(2)#1	3.101(3)	S(4)-Sr(2)#4	3.102(3)
O(1)-Ge(1)-S(1)	115.8(3)	S(4)#11-Sr(1)-S(3)#10	102.24(8)
O(1)-Ge(1)-S(2)	111.7(3)	S(1)#11-Sr(1)-S(3)#10	134.54(9)
S(1)-Ge(1)-S(2)	105.50(12)	S(4)#5-Sr(1)-S(3)#10	93.60(8)
O(1)-Ge(1)-S(3)	111.0(3)	S(3)#9-Sr(1)-S(3)#10	141.95(12)
S(1)-Ge(1)-S(3)	104.50(14)	O(1)-Sr(1)-S(1)#10	122.92(19)
S(2)-Ge(1)-S(3)	107.80(14)	S(4)#11-Sr(1)-S(1)#10	83.25(9)
O(1)-Sr(2)-S(4)	123.2(2)	S(1)#11-Sr(1)-S(1)#10	157.98(6)
O(1)-Sr(2)-S(2)#8	72.72(19)	S(4)#5-Sr(1)-S(1)#10	90.84(9)
S(4)-Sr(2)-S(2)#8	82.11(9)	S(3)#9-Sr(1)-S(1)#10	78.73(8)
O(1)-Sr(2)-S(3)#10	72.0(2)	S(3)#10-Sr(1)-S(1)#10	66.79(8)
S(4)-Sr(2)-S(3)#10	90.95(8)	O(1)-Sr(3)-S(4)#13	127.82(18)
S(2)#8-Sr(2)-S(3)#10	131.93(9)	O(1)-Sr(3)-S(4)#11	71.28(18)
O(1)-Sr(2)-S(4)#11	69.96(19)	S(4)#13-Sr(3)-S(4)#11	159.29(10)
S(4)-Sr(2)-S(4)#11	164.62(12)	O(1)-Sr(3)-S(1)#11	69.52(19)
S(2)#8-Sr(2)-S(4)#11	95.90(9)	S(4)#13-Sr(3)-S(1)#11	94.17(10)
S(3)#10-Sr(2)-S(4)#11	101.49(8)	S(4)#11-Sr(3)-S(1)#11	85.21(10)
O(1)-Sr(2)-S(2)#10	134.31(19)	O(1)-Sr(3)-S(2)#8	70.31(18)
S(4)-Sr(2)-S(2)#10	81.24(10)	S(4)#13-Sr(3)-S(2)#8	98.00(9)
S(2)#8-Sr(2)-S(2)#10	152.97(7)	S(4)#11-Sr(3)-S(2)#8	96.51(9)
S(3)#10-Sr(2)-S(2)#10	69.53(8)	S(1)#11-Sr(3)-S(2)#8	136.80(8)
S(4)#11-Sr(2)-S(2)#10	94.56(10)	O(1)-Sr(3)-S(2)#14	138.79(19)
O(1)-Sr(2)-S(3)#12	128.8(2)	S(4)#13-Sr(3)-S(2)#14	80.44(9)
S(4)-Sr(2)-S(3)#12	95.21(8)	S(4)#11-Sr(3)-S(2)#14	79.05(8)
S(2)#8-Sr(2)-S(3)#12	81.91(9)	S(1)#11-Sr(3)-S(2)#14	80.29(8)
S(3)#10-Sr(2)-S(3)#12	146.16(12)	S(2)#8-Sr(3)-S(2)#14	142.58(10)
S(4)#11-Sr(2)-S(3)#12	69.43(8)	O(1)-Sr(3)-S(1)#8	130.31(19)
S(2)#10-Sr(2)-S(3)#12	78.58(8)	S(4)#13-Sr(3)-S(1)#8	82.98(9)
O(1)-Sr(1)-S(4)#11	71.10(18)	S(4)#11-Sr(3)-S(1)#8	89.18(10)
O(1)-Sr(1)-S(1)#11	70.6(2)	S(1)#11-Sr(3)-S(1)#8	155.90(5)
S(4)#11-Sr(1)-S(1)#11	85.81(9)	S(2)#8-Sr(3)-S(1)#8	67.11(7)
O(1)-Sr(1)-S(4)#5	127.81(19)	S(2)#14-Sr(3)-S(1)#8	75.64(7)
S(4)#11-Sr(1)-S(4)#5	159.20(7)	O(1)-Sr(3)-S(3)	62.18(17)
S(1)#11-Sr(1)-S(4)#5	92.68(9)	S(4)#13-Sr(3)-S(3)	65.74(8)
O(1)-Sr(1)-S(3)#9	146.6(2)	S(4)#11-Sr(3)-S(3)	133.15(8)

S(4)#11-Sr(1)-S(3)#9	88.65(8)	S(1)#11-Sr(3)-S(3)	74.38(8)
S(1)#11-Sr(1)-S(3)#9	81.97(9)	S(2)#8-Sr(3)-S(3)	73.38(8)
S(4)#5-Sr(1)-S(3)#9	70.61(8)	S(2)#14-Sr(3)-S(3)	135.38(9)
O(1)-Sr(1)-S(3)#10	70.2(2)	S(1)#8-Sr(3)-S(3)	124.73(9)
	$Sr_3Se[GeOSe_3]$		
Ge(1)-O(1)	1.756(8)	Sr(1)-Se(4)#3	3.1169(15)
Ge(1)-Se(1)	2.3370(17)	Sr(1)-Se(4)#5	3.1487(15)
Ge(1)-Se(2)	2.3422(18)	Sr(1)-Se(3)#2	3.2737(18)
Ge(1)-Se(3)	2.349(2)	Sr(1)-Se(1)#2	3.302(2)
Sr(2)-O(1)	2.510(8)	Sr(1)-Se(3)#6	3.3219(17)
Sr(2)-Se(4)	3.0553(17)	Sr(1)-Se(2)	3.7919(17)
Sr(2)-Se(2)#1	3.1519(18)	Sr(3)-O(1)	2.602(8)
Sr(2)-Se(3)#2	3.1959(16)	Sr(3)-Se(4)#7	3.0826(19)
Sr(2)-Se(4)#3	3.2302(18)	Sr(3)-Se(4)#3	3.0872(19)
Sr(2)-Se(2)#2	3.2925(19)	Sr(3)-Se(1)#3	3.1785(15)
Sr(2)-Se(3)#4	3.3123(16)	Sr(3)-Se(2)#1	3.2371(17)
Sr(1)-O(1)	2.583(8)	Sr(3)-Se(2)#8	3.3370(17)
Sr(1)-Se(1)#3	3.1056(19)	Sr(3)-Se(1)#1	3.3552(15)
O(1)-Sr(2)-Se(4)	124.87(18)	Se(4)#3-Sr(1)-Se(3)#6	87.69(4)
O(1)-Sr(2)-Se(2)#1	73.86(19)	Se(4)#5-Sr(1)-Se(3)#6	68.15(4)
Se(4)-Sr(2)-Se(2)#1	80.87(4)	Se(3)#2-Sr(1)-Se(3)#6	140.89(5)
O(1)-Sr(2)-Se(3)#2	73.57(19)	Se(1)#2-Sr(1)-Se(3)#6	76.86(4)
Se(4)-Sr(2)-Se(3)#2	91.08(4)	O(1)-Sr(3)-Se(4)#7	130.98(17)
Se(2)#1-Sr(2)-Se(3)#2	133.10(5)	O(1)-Sr(3)-Se(4)#3	71.06(17)
O(1)-Sr(2)-Se(4)#3	69.62(18)	Se(4)#7-Sr(3)-Se(4)#3	156.12(5)
Se(4)-Sr(2)-Se(4)#3	162.85(5)	O(1)-Sr(3)-Se(1)#3	70.42(18)
Se(2)#1-Sr(2)-Se(4)#3	96.37(4)	Se(4)#7-Sr(3)-Se(1)#3	94.48(5)
Se(3)#2-Sr(2)-Se(4)#3	102.82(4)	Se(4)#3-Sr(3)-Se(1)#3	84.42(5)
O(1)-Sr(2)-Se(2)#2	137.13(19)	O(1)-Sr(3)-Se(2)#1	71.25(18)
Se(4)-Sr(2)-Se(2)#2	79.93(4)	Se(4)#7-Sr(3)-Se(2)#1	98.91(4)
Se(2)#1-Sr(2)-Se(2)#2	148.95(4)	Se(4)#3-Sr(3)-Se(2)#1	97.53(4)
Se(3)#2-Sr(2)-Se(2)#2	71.47(4)	Se(1)#3-Sr(3)-Se(2)#1	138.59(4)
Se(4)#3-Sr(2)-Se(2)#2	94.85(4)	O(1)-Sr(3)-Se(2)#8	137.64(17)
O(1)-Sr(2)-Se(3)#4	126.41(19)	Se(4)#7-Sr(3)-Se(2)#8	78.85(4)
Se(4)-Sr(2)-Se(3)#4	95.51(4)	Se(4)#3-Sr(3)-Se(2)#8	77.52(4)
Se(2)#1-Sr(2)-Se(3)#4	80.72(4)	Se(1)#3-Sr(3)-Se(2)#8	79.05(4)
Se(3)#2-Sr(2)-Se(3)#4	146.19(6)	Se(2)#1-Sr(3)-Se(2)#8	141.95(5)
Se(4)#3-Sr(2)-Se(3)#4	67.35(4)	O(1)-Sr(3)-Se(1)#1	132.10(18)
Se(2)#2-Sr(2)-Se(3)#4	77.11(4)	Se(4)#7-Sr(3)-Se(1)#1	80.81(4)
O(1)-Sr(1)-Se(1)#3	71.89(18)	Se(4)#3-Sr(3)-Se(1)#1	89.21(4)
O(1)-Sr(1)-Se(4)#3	70.78(17)	Se(1)#3-Sr(3)-Se(1)#1	152.57(4)
Se(1)#3-Sr(1)-Se(4)#3	85.16(4)	Se(2)#1-Sr(3)-Se(1)#1	68.67(3)
O(1)-Sr(1)-Se(4)#5	131.37(18)	Se(2)#8-Sr(3)-Se(1)#1	73.53(3)
Se(1)#3-Sr(1)-Se(4)#5	92.76(4)	O(1)-Sr(3)-Se(3)	65.02(17)
Se(4)#3-Sr(1)-Se(4)#5	155.75(4)	Se(4)#7-Sr(3)-Se(3)	66.08(4)
O(1)-Sr(1)-Se(3)#2	71.33(19)	Se(4)#3-Sr(3)-Se(3)	135.60(4)
Se(1)#3-Sr(1)-Se(3)#2	136.56(5)	Se(1)#3-Sr(3)-Se(3)	75.43(4)
Se(4)#3-Sr(1)-Se(3)#2	103.60(4)	Se(2)#1-Sr(3)-Se(3)	74.72(4)
Se(4)#5-Sr(1)-Se(3)#2	94.50(4)	Se(2)#8-Sr(3)-Se(3)	134.15(4)
O(1)-Sr(1)-Se(1)#2	122.79(18)	Se(1)#1-Sr(3)-Se(3)	125.24(5)
Se(1)#3-Sr(1)-Se(1)#2	154.26(4)	O(1)-Ge(1)-Se(1)	116.8(3)

Se(4)#3-Sr(1)-Se(1)#2	81.17(4)	O(1)-Ge(1)-Se(2)	111.6(3)
Se(4)#5-Sr(1)-Se(1)#2	90.89(4)	Se(1)-Ge(1)-Se(2)	105.30(6)
Se(3)#2-Sr(1)-Se(1)#2	68.35(4)	O(1)-Ge(1)-Se(3)	110.6(3)
O(1)-Sr(1)-Se(3)#6	146.25(19)	Se(1)-Ge(1)-Se(3)	104.04(7)
Se(1)#3-Sr(1)-Se(3)#6	80.90(4)	Se(2)-Ge(1)-Se(3)	107.81(8)
	$Ba_3S[GeOS_3]$		
Ge(1)-O(1)	1.755(7)	Ba(2)-S(4)	3.057(3)
Ge(1)-S(1)	2.207(3)	Ba(2)-S(3)#6	3.230(3)
Ge(1)-S(2)	2.223(3)	Ba(2)-S(4)#1	3.254(3)
Ge(1)-S(3)	2.221(3)	Ba(2)-S(2)#3	3.267(3)
Ba(1)-O(1)	2.706(8)	Ba(2)-S(3)#3	3.342(3)
Ba(1)-S(4)#5	3.095(3)	Ba(2)-S(2)#9	3.369(3)
Ba(1)-S(4)#1	3.121(3)	Ba(3)-O(1)	2.684(8)
Ba(1)-S(1)#1	3.271(3)	Ba(3)-S(4)#1	3.148(3)
Ba(1)-S(3)#6	3.315(3)	Ba(3)-S(4)#2	3.161(3)
Ba(1)-S(3)#7	3.381(3)	Ba(3)-S(1)#1	3.190(4)
Ba(1)-S(1)#6	3.388(3)	Ba(3)-S(2)#3	3.354(3)
Ba(1)-S(2)	3.529(3)	Ba(3)-S(2)#4	3.360(3)
Ba(2)-O(1)	2.616(8)	Ba(3)-S(1)#3	3.362(4)
O(1)-Ba(3)-S(4)#1	69.95(16)	S(4)#1-Ba(1)-S(1)#6	89.70(8)
O(1)-Ba(3)-S(4)#2	130.17(17)	S(1)#1-Ba(1)-S(1)#6	153.59(4)
S(4)#1-Ba(3)-S(4)#2	157.46(5)	S(3)#6-Ba(1)-S(1)#6	64.36(7)
O(1)-Ba(3)-S(1)#1	72.86(17)	S(3)#7-Ba(1)-S(1)#6	74.44(7)
S(4)#1-Ba(3)-S(1)#1	83.71(8)	O(1)-Ba(1)-S(2)	61.42(16)
S(4)#2-Ba(3)-S(1)#1	92.67(8)	S(4)#5-Ba(1)-S(2)	67.67(7)
O(1)-Ba(3)-S(2)#3	72.12(17)	S(4)#1-Ba(1)-S(2)	131.08(7)
S(4)#1-Ba(3)-S(2)#3	101.58(8)	S(1)#1-Ba(1)-S(2)	76.17(8)
S(4)#2-Ba(3)-S(2)#3	95.46(7)	S(3)#6-Ba(1)-S(2)	76.37(7)
S(1)#1-Ba(3)-S(2)#3	140.07(8)	S(3)#7-Ba(1)-S(2)	137.94(8)
O(1)-Ba(3)-S(2)#4	147.73(17)	S(1)#6-Ba(1)-S(2)	126.00(8)
S(4)#1-Ba(3)-S(2)#4	88.27(7)	O(1)-Ba(2)-S(4)	122.70(18)
S(4)#2-Ba(3)-S(2)#4	69.19(7)	O(1)-Ba(2)-S(3)#6	74.47(18)
S(1)#1-Ba(3)-S(2)#4	81.57(8)	S(4)-Ba(2)-S(3)#6	81.08(8)
S(2)#3-Ba(3)-S(2)#4	137.58(10)	O(1)-Ba(2)-S(4)#1	68.96(17)
O(1)-Ba(3)-S(1)#3	120.97(17)	S(4)-Ba(2)-S(4)#1	165.08(10)
S(4)#1-Ba(3)-S(1)#3	82.64(8)	S(3)#6-Ba(2)-S(4)#1	94.53(8)
S(4)#2-Ba(3)-S(1)#3	92.03(8)	O(1)-Ba(2)-S(2)#3	74.41(18)
S(1)#1-Ba(3)-S(1)#3	155.12(4)	S(4)-Ba(2)-S(2)#3	91.54(7)
S(2)#3-Ba(3)-S(1)#3	63.51(7)	S(3)#6-Ba(2)-S(2)#3	136.97(8)
S(2)#4-Ba(3)-S(1)#3	77.33(7)	S(4)#1-Ba(2)-S(2)#3	101.20(7)
O(1)-Ba(1)-S(4)#5	129.03(17)	O(1)-Ba(2)-S(3)#3	134.18(18)
O(1)-Ba(1)-S(4)#1	70.12(16)	S(4)-Ba(2)-S(3)#3	82.46(9)
S(4)#5-Ba(1)-S(4)#1	159.01(9)	S(3)#6-Ba(2)-S(3)#3	151.28(6)
O(1)-Ba(1)-S(1)#1	71.25(17)	S(4)#1-Ba(2)-S(3)#3	95.38(8)
S(4)#5-Ba(1)-S(1)#1	95.04(8)	S(2)#3-Ba(2)-S(3)#3	66.67(7)
S(4)#1-Ba(1)-S(1)#1	82.80(8)	O(1)-Ba(2)-S(2)#9	127.65(17)
O(1)-Ba(1)-S(3)#6	71.94(17)	S(4)-Ba(2)-S(2)#9	97.14(8)
S(4)#5-Ba(1)-S(3)#6	99.06(7)	S(3)#6-Ba(2)-S(2)#9	80.63(7)
S(4)#1-Ba(1)-S(3)#6	95.41(7)	S(4)#1-Ba(2)-S(2)#9	68.02(7)
S(1)#1-Ba(1)-S(3)#6	141.38(7)	S(2)#3-Ba(2)-S(2)#9	142.38(10)
O(1)-Ba(1)-S(3)#7	138.40(17)	S(3)#3-Ba(2)-S(2)#9	78.26(7)

S(4)#5-Ba(1)-S(3)#7	81.27(7)	O(1)-Ge(1)-S(1)	114.6(3)
S(4)#1-Ba(1)-S(3)#7	77.81(7)	O(1)-Ge(1)-S(3)	109.6(3)
S(1)#1-Ba(1)-S(3)#7	79.22(7)	S(1)-Ge(1)-S(3)	107.48(12)
S(3)#6-Ba(1)-S(3)#7	138.33(9)	O(1)-Ge(1)-S(2)	109.6(3)
O(1)-Ba(1)-S(1)#6	129.56(17)	S(1)-Ge(1)-S(2)	105.85(14)
S(4)#5-Ba(1)-S(1)#6	83.01(8)	S(3)-Ge(1)-S(2)	109.63(13)
	$Ba_3Se[GeOSe_3]$		
Se(1)-Ge(1)	2.343(3)	Se(3)-Ba(1)#6	3.515(2)
Se(1)-Ba(1)#1	3.290(2)	Se(3)-Ba(2)#3	3.521(2)
Se(1)-Ba(3)#2	3.393(2)	Se(3)-Ba(3)#3	3.530(2)
Se(1)-Ba(1)#3	3.478(2)	Ge(1)-O(1)	1.751(11)
Se(1)-Ba(3)	3.514(2)	O(1)-Ba(2)#7	2.641(12)
Se(2)-Ge(1)	2.357(3)	O(1)-Ba(1)#4	2.722(11)
Se(2)-Ba(2)#4	3.331(2)	O(1)-Ba(3)#3	2.738(12)
Se(2)-Ba(3)	3.433(2)	Se(4)-Ba(2)#8	3.177(2)
Se(2)-Ba(2)	3.469(2)	Se(4)-Ba(3)	3.197(2)
Se(2)-Ba(3)#5	3.519(2)	Se(4)-Ba(3)#9	3.219(2)
Se(2)-Ba(1)#4	3.744(2)	Se(4)-Ba(1)#3	3.262(2)
Se(3)-Ge(1)	2.358(3)	Se(4)-Ba(1)#8	3.300(2)
Se(3)-Ba(2)	3.352(2)	Se(4)-Ba(2)	3.390(2)
Se(3)-Ba(1)#3	3.454(2)	O(1)-Ge(1)-Se(1)	115.3(4)
O(1)-Ge(1)-Se(2)	109.6(4)	Se(2)#9-Ba(2)-Se(4)	94.89(5)
Se(1)-Ge(1)-Se(2)	107.23(10)	Se(3)-Ba(2)-Se(4)	102.34(5)
O(1)-Ge(1)-Se(3)	109.3(4)	O(1)#14-Ba(2)-Se(2)	136.7(2)
Se(1)-Ge(1)-Se(3)	105.76(10)	Se(4)#5-Ba(2)-Se(2)	81.13(5)
Se(2)-Ge(1)-Se(3)	109.46(10)	Se(2)#9-Ba(2)-Se(2)	147.59(4)
O(1)#9-Ba(1)-Se(4)#10	69.9(2)	Se(3)-Ba(2)-Se(2)	68.70(5)
O(1)#9-Ba(1)-Se(1)#12	73.7(3)	Se(4)-Ba(2)-Se(2)	95.21(5)
Se(4)#10-Ba(1)-Se(1)#12	83.29(5)	O(1)#14-Ba(2)-Se(3)#10	124.6(3)
O(1)#9-Ba(1)-Se(4)#5	133.2(2)	Se(4)#5-Ba(2)-Se(3)#10	97.39(5)
Se(4)#10-Ba(1)-Se(4)#5	154.15(4)	Se(2)#9-Ba(2)-Se(3)#10	79.26(5)
Se(1)#12-Ba(1)-Se(4)#5	92.27(5)	Se(3)-Ba(2)-Se(3)#10	142.65(6)
O(1)#9-Ba(1)-Se(3)#10	73.1(3)	Se(4)-Ba(2)-Se(3)#10	65.45(4)
Se(4)#10-Ba(1)-Se(3)#10	102.86(5)	Se(2)-Ba(2)-Se(3)#10	77.16(5)
Se(1)#12-Ba(1)-Se(3)#10	141.57(5)	O(1)#10-Ba(3)-Se(4)	130.8(2)
Se(4)#5-Ba(1)-Se(3)#10	96.42(5)	O(1)#10-Ba(3)-Se(4)#4	70.4(2)
O(1)#9-Ba(1)-Se(1)#10	121.6(3)	Se(4)-Ba(3)-Se(4)#4	156.79(6)
Se(4)#10-Ba(1)-Se(1)#10	81.02(5)	O(1)#10-Ba(3)-Se(1)#15	71.8(2)
Se(1)#12-Ba(1)-Se(1)#10	151.72(4)	Se(4)-Ba(3)-Se(1)#15	95.12(5)
Se(4)#5-Ba(1)-Se(1)#10	91.70(5)	Se(4)#4-Ba(3)-Se(1)#15	82.33(5)
Se(3)#10-Ba(1)-Se(1)#10	65.47(4)	O(1)#10-Ba(3)-Se(2)	72.6(2)
O(1)#9-Ba(1)-Se(3)#13	147.0(3)	Se(4)-Ba(3)-Se(2)	99.58(5)
Se(4)#10-Ba(1)-Se(3)#13	87.71(5)	Se(4)#4-Ba(3)-Se(2)	96.15(5)
Se(1)#12-Ba(1)-Se(3)#13	80.01(5)	Se(1)#15-Ba(3)-Se(2)	142.61(5)
Se(4)#5-Ba(1)-Se(3)#13	66.44(5)	O(1)#10-Ba(3)-Se(1)	131.4(3)
Se(3)#10-Ba(1)-Se(3)#13	137.43(6)	Se(4)-Ba(3)-Se(1)	81.36(5)
Se(1)#10-Ba(1)-Se(3)#13	76.00(4)	Se(4)#4-Ba(3)-Se(1)	89.66(5)
O(1)#9-Ba(1)-Se(2)#9	60.5(2)	Se(1)#15-Ba(3)-Se(1)	150.81(4)
Se(4)#10-Ba(1)-Se(2)#9	128.87(5)	Se(2)-Ba(3)-Se(1)	65.98(4)
Se(1)#12-Ba(1)-Se(2)#9	72.36(5)	O(1)#10-Ba(3)-Se(2)#8	137.7(2)
Se(4)#5-Ba(1)-Se(2)#9	72.75(5)	Se(4)-Ba(3)-Se(2)#8	80.08(4)

Se(3)#10-Ba(1)-Se(2)#9	74.71(4)	Se(4)#4-Ba(3)-Se(2)#8	76.82(4)
Se(1)#10-Ba(1)-Se(2)#9	135.24(5)	Se(1)#15-Ba(3)-Se(2)#8	78.15(5)
Se(3)#13-Ba(1)-Se(2)#9	129.08(5)	Se(2)-Ba(3)-Se(2)#8	138.14(6)
O(1)#14-Ba(2)-Se(4)#5	124.9(2)	Se(1)-Ba(3)-Se(2)#8	72.69(4)
O(1)#14-Ba(2)-Se(2)#9	75.4(2)	O(1)#10-Ba(3)-Se(3)#10	63.6(2)
Se(4)#5-Ba(2)-Se(2)#9	80.19(5)	Se(4)-Ba(3)-Se(3)#10	67.31(4)
O(1)#14-Ba(2)-Se(3)	75.9(3)	Se(4)#4-Ba(3)-Se(3)#10	133.36(5)
Se(4)#5-Ba(2)-Se(3)	92.03(5)	Se(1)#15-Ba(3)-Se(3)#10	76.88(5)
Se(2)#9-Ba(2)-Se(3)	138.07(5)	Se(2)-Ba(3)-Se(3)#10	77.50(4)
O(1)#14-Ba(2)-Se(4)	68.6(2)	Se(1)-Ba(3)-Se(3)#10	126.53(5)
Se(4)#5-Ba(2)-Se(4)	162.81(7)	Se(2)#8-Ba(3)-Se(3)#10	136.41(5)

Symmetry transformations used to generate equivalent atoms for $Sr_3S[GeOS_3]$: #1 -x,-y,z-1/2 #2 -x,-y+1,z-1/2 #3 -x+1/2,y,z-1/2 #4 x,y-1,z #5 x-1/2,-y,z #6 x-1/2,-y+1,z #7 -x+1/2,y-1,z+1/2 #8 x+1/2,-y,z #9 -x,-y+1,z+1/2 #10 -x,-y,z+1/2 #11 x,y+1,z #12 -x+1/2,y,z+1/2 #13 -x+1/2,y+1,z-1/2 #14 x+1/2,-y+1,z.

Symmetry transformations used to generate equivalent atoms for $Sr_3Se[GeOSe_3]$: #1 x-1/2,-y+1,z #2 -x+1,-y+1,z+1/2 #3 x,y-1,z #4 -x+1/2,y,z+1/2 #5 x+1/2,-y+1,z #6 -x+1,-y,z+1/2 #7 -x+1/2,y-1,z-1/2 #8 x-1/2,-y,z #9 -x+1/2,y,z-1/2 #10 -x+1,-y+1,z-1/2 #11 -x+1,-y,z-1/2 #12 -x+1/2,y+1,z+1/2 #13 x,y+1,z #14 x+1/2,-y,z.

Symmetry transformations used to generate equivalent atoms for $Ba_3S[GeOS_3]$: #1 x,y+1,z #2 x+1/2,-y+1,z #3 -x+1,-y+1,z+1/2 #4 -x+1,-y+2,z+1/2 #5 -x+1/2,y+1,z-1/2 #6 x-1/2,-y+1,z #7 x-1/2,-y+2,z #8 -x+1/2,y,z-1/2 #9 -x+1/2,y,z+1/2 #10 x,y-1,z #11 -x+1/2,y-1,z+1/2 #12 -x+1,-y+1,z-1/2 #13 -x+1,-y+2,z-1/2 #14 x+1/2,-y+2,z.

Symmetry transformations used to generate equivalent atoms for $Ba_3Se[GeOSe_3]$: #1 -x+1/2,y-1,z-1/2 #2 x+1/2,-y-1,z #3 x+1/2,-y,z #4 -x+1/2,y,z-1/2 #5 x,y+1,z #6 x+1/2,-y+1,z #7 -x+1,-y,z-1/2 #8 x,y-1,z #9 -x+1/2,y,z+1/2 #10 x-1/2,-y,z #11 -x,-y,z+1/2 #12 -x+1/2,y+1,z+1/2 #13 x-1/2,-y+1,z #14 -x+1,-y,z+1/2 #15 x-1/2,-y-1,z #16 -x,-y,z-1/2.

Table S3a. atomic coordinates (× 10^4) and equivalent isotropic displacement parameters ($Å^2$ × 10^3) for $Sr_3S[GeOS_3]$. U_{eq} is defined as one-third of the trace of the orthogonalized U_{ij} tensor.

Atom	*Wyck.*	*x*	*y*	*z*	U_{eq}	BVS[a)]
Sr(1)	4a	-186(1)	4161(2)	6974(1)	10(1)	1.91
Sr(2)	4a	2003(1)	-354(2)	7038(1)	10(1)	1.97
Sr(3)	4a	2299(1)	3987(2)	4631(1)	11(1)	1.98
Ge(1)	4a	-42(1)	77(3)	4571(1)	8(1)	4.11
O(1)	4a	787(7)	1513(13)	5569(7)	11(2)	1.96
S(1)	4a	18(2)	-3601(5)	4675(3)	12(1)	2.04
S(2)	4a	-1849(2)	967(5)	4727(3)	11(1)	1.97
S(3)	4a	497(3)	847(5)	2800(3)	11(1)	1.91
S(4)	4a	2325(2)	-5213(4)	7152(3)	10(1)	2.00

[a)]Bond valence state was obtained by the empirical formula $V_i = \Sigma S_{ij} = \Sigma \exp[(r_0 - r_{ij})/0.37]$, where S_{ij} is the bond valence associated with bond lengths r_{ij} and r_0.[1]

Table S3b. atomic coordinates (× 10^4) and equivalent isotropic displacement parameters ($Å^2$ × 10^3) for $Sr_3Se[GeOSe_3]$. U_{eq} is defined as one-third of the trace of the orthogonalized U_{ij} tensor.

Atom	*Wyck.*	x	y	z	U(eq)	BVS[a)]
Sr(1)	4a	5227(1)	973(2)	6963(1)	11(1)	1.92
Sr(2)	4a	3077(1)	5386(2)	7052(1)	11(1)	2.00
Sr(3)	4a	2736(1)	1159(2)	4656(1)	11(1)	2.03
Ge(1)	4a	5021(1)	4882(2)	4626(1)	8(1)	4.04
O(1)	4a	4219(7)	3521(13)	5611(7)	11(2)	1.88
Se(1)	4a	4996(1)	8640(2)	4692(1)	12(1)	2.07
Se(2)	4a	6867(1)	3926(2)	4766(1)	12(1)	2.04
Se(3)	4a	4436(1)	4068(2)	2814(1)	11(1)	2.01
Se(4)	4a	2716(1)	10244(2)	7178(1)	10(1)	2.06

[a)]Bond valence state was obtained by the empirical formula $V_i = \Sigma S_{ij} = \Sigma \exp[(r_0 - r_{ij})/0.37]$, where S_{ij} is the bond valence associated with bond lengths r_{ij} and r_0.[1]

Table S3c. atomic coordinates (× 10^4) and equivalent isotropic displacement parameters ($Å^2$ × 10^3) for $Ba_3S[GeOS_3]$. U_{eq} is defined as one-third of the trace of the orthogonalized U_{ij} tensor.

Atom	*Wyck.*	x	y	z	U(eq)	BVS[a)]
Ba(1)	4a	2694(1)	8883(1)	4579(1)	11(1)	2.12
Ba(2)	4a	3045(1)	4542(1)	7033(1)	12(1)	2.10
Ba(3)	4a	5245(1)	9078(1)	6930(1)	11(1)	1.98
Ge(1)	4a	5026(1)	5092(2)	4560(1)	9(1)	3.98
O(1)	4a	4229(6)	6426(12)	5537(6)	12(2)	2.06
S(1)	4a	4962(2)	1574(5)	4664(3)	15(1)	2.00
S(2)	4a	4456(2)	5917(5)	2880(2)	13(1)	1.98
S(3)	4a	6745(2)	6061(5)	4752(3)	13(1)	1.91
S(4)	4a	2722(2)	-299(4)	7107(3)	11(1)	1.97

a)Bond valence state was obtained by the empirical formula $V_i = \Sigma S_{ij} = \Sigma \exp[(r_0 - r_{ij})/0.37]$, where S_{ij} is the bond valence associated with bond lengths r_{ij} and r_0.[1]

Table S3d. atomic coordinates (× 10^4) and equivalent isotropic displacement parameters (Å^2 × 10^3) for $Ba_3Se[GeOSe_3]$. U_{eq} is defined as one-third of the trace of the orthogonalized U_{ij} tensor.

Atom	*Wyck.*	x	y	z	U(eq)	BVS[a)]
Ba(1)	4a	271(1)	3947(2)	3078(1)	15(1)	2.01
Ba(2)	4a	3120(1)	516(1)	2965(1)	16(1)	2.04
Ba(3)	4a	2280(1)	-3758(2)	396(1)	14(1)	2.13
Ge(1)	4a	4998(2)	138(3)	400(2)	11(1)	3.97
O(1)	4a	5768(9)	1398(18)	-558(9)	13(2)	1.99
Se(1)	4a	5029(2)	-3466(3)	319(2)	18(1)	1.98
Se(2)	4a	3237(1)	1172(3)	215(1)	16(1)	1.98
Se(3)	4a	5602(1)	1002(3)	2124(1)	14(1)	2.00
Se(4)	4a	2748(1)	-4651(3)	2864(2)	16(1)	2.20

[a)]Bond valence state was obtained by the empirical formula $V_i = \Sigma S_{ij} = \Sigma \exp[(r_0 - r_{ij})/0.37]$, where S_{ij} is the bond valence associated with bond lengths r_{ij} and r_0.[1]

Table S4. Summary of the tolerance factor, distortion index , bond angle variance and volume of site group.

Compound	Tolerance factor (*t*)	Distortion index (*D*)	Bond angle variance (σ^2)	Volume of A-site group
$Ba_3S[GeOS_3]$	0.85	0.0154	112.9	4.72
$Ba_3Se[GeOSe_3]$	0.82	0.0184	144.2	5.41
$Sr_3S[GeOS_3]$	0.86	0.0134	103.0	4.66
$Sr_3Se[GeOSe_3]$	0.83	0.0148	135.9	5.36
$Ba_3S[GeS_4]$	0.77	0.0214	56.3	5.37

Table S5. The Global Instability Index (GII) of title compounds and Ba_3GeS_5.

Compound	GII
$Ba_3S[GeS_4]$	0.41
$Sr_3S[GeOS_3]$	0.05
$Sr_3Se[GeOSe_3]$	0.06
$Ba_3S[GeOS_3]$	0.11
$Ba_3Se[GeOSe_3]$	0.08

The global instability index (GII) is often used to evaluate the rationality of the structure. In order to guarantee the rationality of crystal structures of title compounds, the GII values were calculated. When the value of GII is less than 0.05 vu (valence unit), the tension of structure is not proper, whereas the value of GII is larger than 0.2 vu, its structure is not stable.[2] As for title compounds, calculated GII values are in the range of 0.05−0.11 vu, indicating that the crystal structures of all compounds are reasonable.

Table S6. Dipole moments (in Debye) and magnitudes of out-of-center distortions of [QAe_6] and [$GeOQ_3$] (Ae = Sr, Ba; Q = S, Se) polyhedra in the unit cell.

Compound	SBUs	x	y	z	Ge-O (D)	Ge-Q (D)	Dipole moment(D)	Δd
$Sr_3S[GeOS_3]$	$GeOS_3$	-6.44	-5.30	8.28	13.59	25.34	11.76	-
	SSr_6	-2.74	-5.74	-5.21	-	-	8.22	0.21
$Sr_3Se[GeOSe_3]$	$GeOSe_3$	-11.24	8.91	-14.76	13.43	33.98	20.58	-
	$SeSr_6$	2.44	-5.10	-5.21	-	-	7.69	0.22
$Ba_3S[GeOS_3]$	$GeOS_3$	-5.33	-4.24	-6.98	13.32	23.06	9.75	-
	SBa_6	-2.69	-6.83	-5.85	-	-	9.39	0.25
$Ba_3Se[GeOSe_3]$	$GeOSe_3$	-9.90	7.34	13.00	13.34	31.23	17.91	-
	$SeBa_6$	-2.80	-5.68	6.09	-	-	8.78	0.29
$Ba_3S[GeS_4]$	GeS_4	0.62	0	-0.70	22.36	22.86	0.94	0.45
	SBa_6	3.06	0	4.52	-	-	5.46	

Table S7. Property comparison of title compounds with other reported oxychalcogenides

Style	Compound	SBUs	SHG	Eg(eV)
Ln-based-type	$LaCaGa_3S_6O$[3]	$(La/Ca)OS_7$, $GaOS_3$	0.9 × AGS	3.27
	$LaSrGa_3S_6O$[3]	$(La/Sr)OS_7$, $GaOS_3$	1.0 × AGS	3.21
$AeGeOQ_2$-type	$SrGeOS_2$[4]	GeO_2S_2	0.4 × AGS	3.90
	$BaGeOS_2$[4]	GeO_2S_2	0.5 × AGS	4.10
	$BaGeOSe_2$[5]	GeO_2Se_2	1.1 × AGS	3.20
	$SrGeOSe_2$[6]	GeO_2Se_2	1.3 × AGS	3.16
melilite-type	$Sr_2MnGe_2OS_6$[7]	$GeOS_3$, MnS_4	0.3 × AGS	3.51
	$Sr_2ZnGe_2OS_6$[7]	$GeOS_3$, ZnS_4	0.6 × AGS	3.73
	$Sr_2ZnSn_2OS_6$[8]	$SnOS_3$, ZnS_4	0.7 × AGS	3.52
	$Sr_2CdGe_2OS_6$[7]	$GeOS_3$, CdS_4	0.8 × AGS	3.52
	$Sr_3Ge_2O_4Se_3$[9]	$GeOSe_3$, GeO_4	0.8 × AGS	2.96
	$Sr_5Ga_8O_3S_{14}$[10]	$GaOS_3$, GaS_4	0.8 × AGS	3.9
	$Ba_3Ge_2O_4Te_3$[11]	$GeOTe_3$, GeO_4	0.6 × AGSe	2.10
	$Sr_2GeGa_2OS_6$[12]	$(Ga/Ge)OS_3$, GaS_4	1.7 × AGS	3.15
	$Ca_2GeGa_2OS_6$[12]	$(Ga/Ge)OS_3$, GaS_4	2.1 × AGS	3.15
Miscellaneous Materials	$Ba_2SnSSi_2O_7$[13]	SnO_4S	2.0 × SiO_2	2.7
	$SrZn_2OS_2$[14]	ZnS_3O	2.0 × KDP	3.86
	$CaZnOS$[15]	ZnS_3O	100 × SiO_2	3.71
	$Sr_3[SnOSe_3][CO_3]$[16]	$SnOSe_3$, CO_3	1.0 × AGS	3.46
	$Sr_6Cd_2Sb_6O_7S_{10}$[17]	SbS_5, $SbOS_4$	4.0 × AGS	1.89
antiperovskite-type	$Ba_3S[GeOS_3]$	SBa_6, $GeOS_3$	0.7 × AGS	3.63
	$Sr_3S[GeOS_3]$	SSr_6, $GeOS_3$	1.0 × AGS	4.10
	$Ba_3Se[GeOSe_3]$	$SeBa_6$, $GeOSe_3$	1.2 × AGS	3.52
	$Sr_3Se[GeOSe_3]$	$SeSr_6$, $GeOSe_3$	1.5 × AGS	3.52

Table S8. Contrast of the normalized dipole moments for mixed-anion SBUs.

Compound	SBUs	Dipole moment (D)	Normalization (D/Å^3)[b]
$Sr_3Se[GeOSe_3]$[a]	$GeOSe_3$	20.58	0.0225
$SrGeOSe_2$	GeO_2Se_2	10.68	0.0222
$BaGeOSe_2$	GeO_2Se_2	11.25	0.0217
$Ba_3Se[GeOSe_3]$[a]	$GeOSe_3$	17.91	0.0173
$Sr_3S[GeOS_3]$[a]	$GeOS_3$	11.76	0.0142
$Sr_3Ge_2O_4Se_3$	$GeOSe_3$	10.17	0.0132
$BaGeOS_2$	GeO_2S_2	5.13	0.0104
$Ba_3S[GeOS_3]$[a]	$GeOS_3$	9.75	0.0104
$SrGeOS_2$	GeO_2S_2	2.96	0.0065
$Ba_3Ge_2O_4Te_3$	$GeOTe_3$	18.40	0.0197

[a] This work.

[b] The normalization dipole moment is equal to the dipole moment of the tetrahedron/unit cell volume.

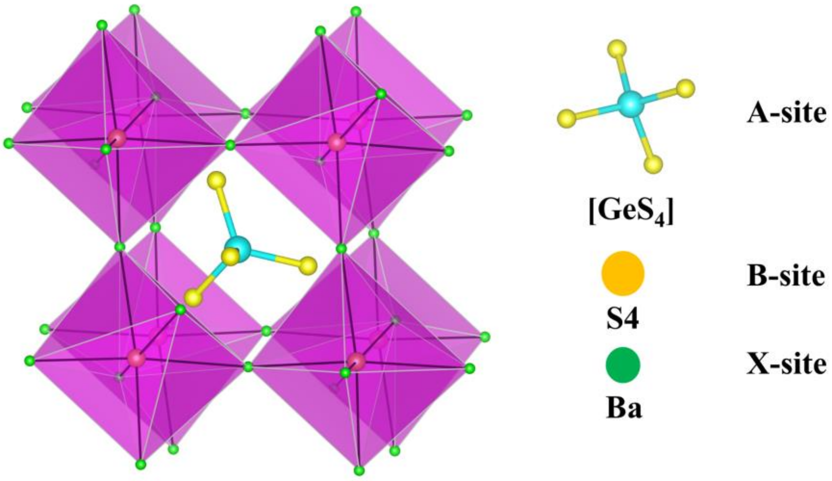

Figure S1. The antiperovskite-type $Ba_3S[GeS_4]$ structure.

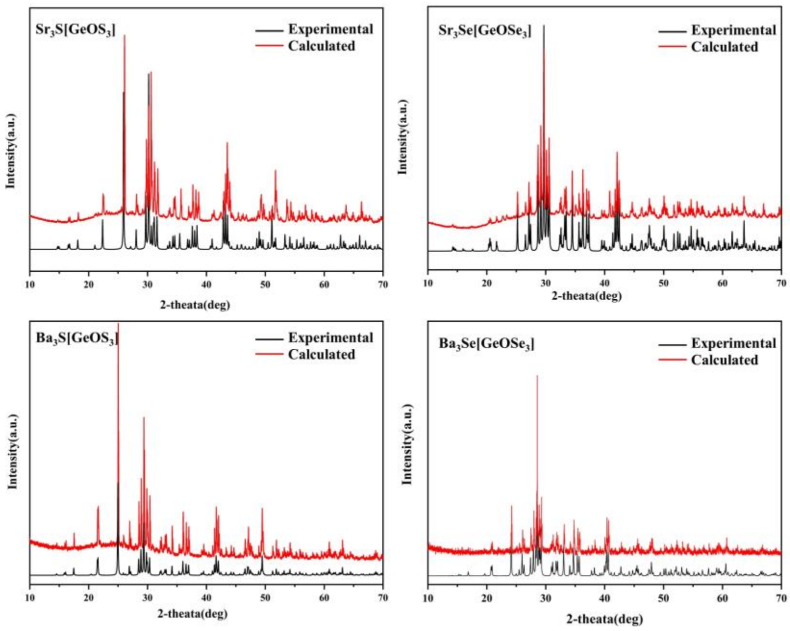

Figure S2. Experimental and standard XRD patterns of title compounds.

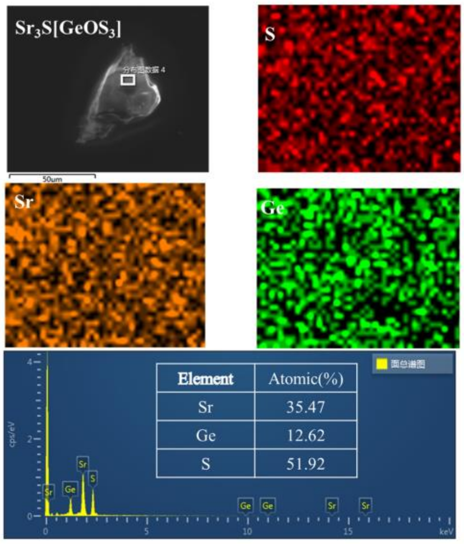

Figure S3a. The energy dispersive spectroscopy of $Sr_3S[GeOS_3]$.

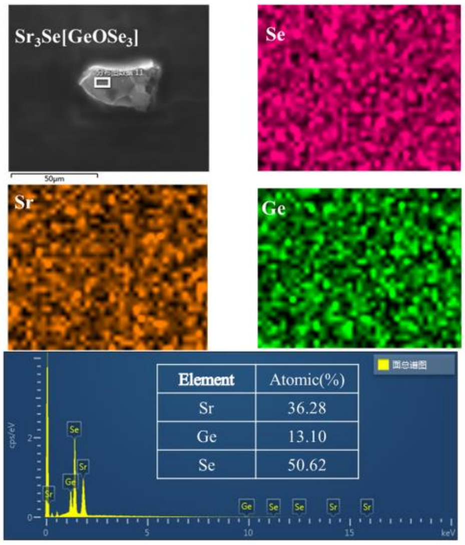

Figure S3b. The energy dispersive spectroscopy of $Sr_3Se[GeOSe_3]$.

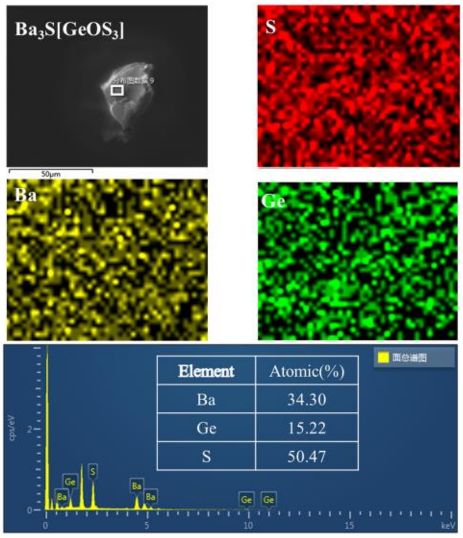

Figure S3c. The energy dispersive spectroscopy of $Ba_3S[GeOS_3]$.

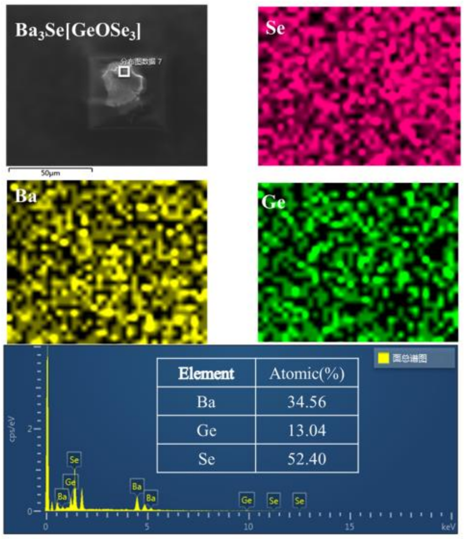

Figure S3d. The energy dispersive spectroscopy of $Ba_3Se[GeOSe_3]$.

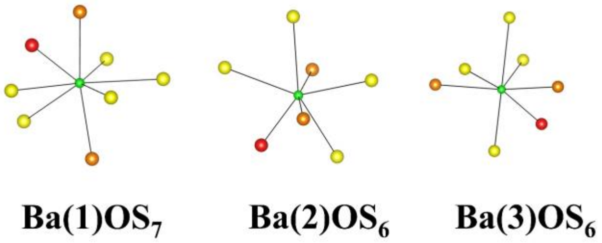

Figure S4. The coordination environment of the Ba atoms in $Ba_3S[GeOS_3]$.

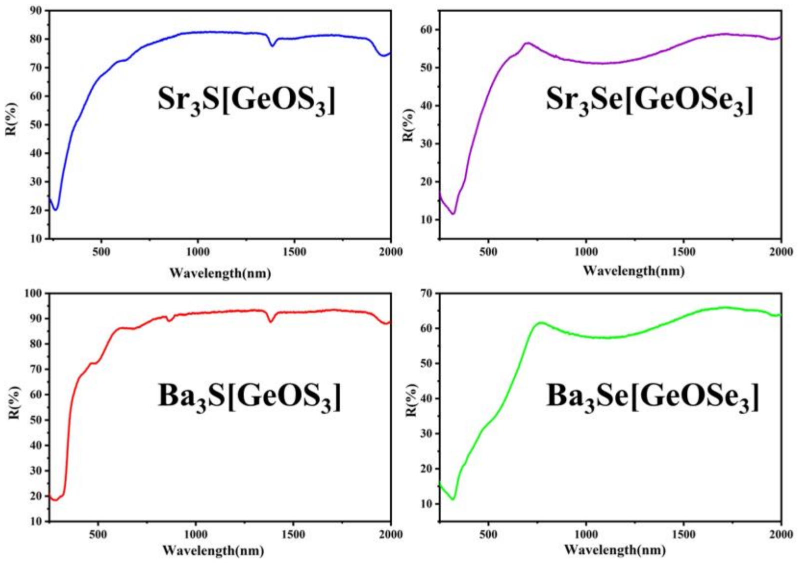

Figure S5. UV-Vis diffuse reflectance spectra of title compounds.

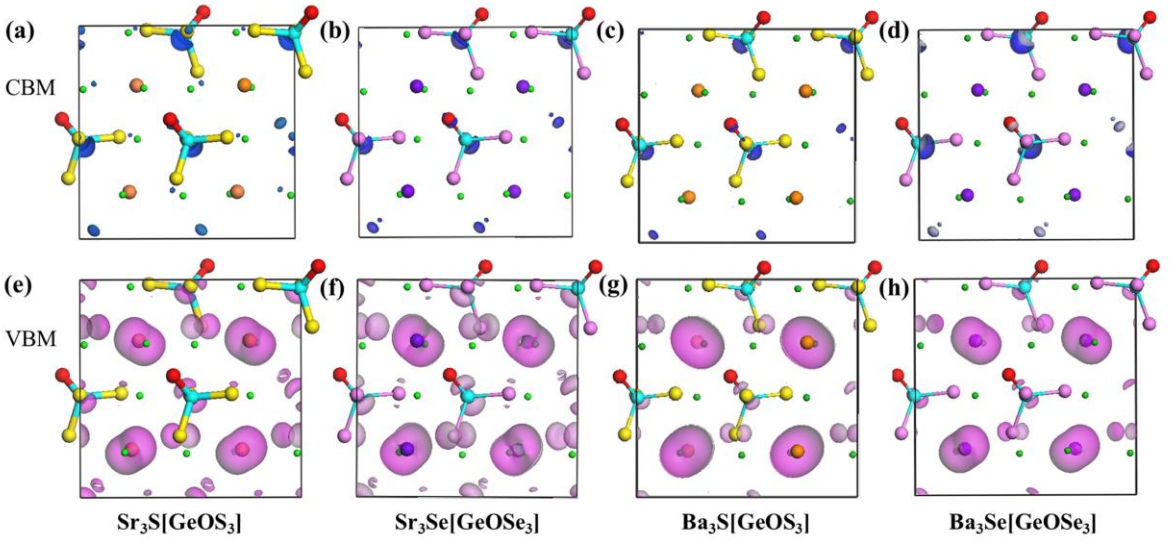

Figure S6. Plots of the charge densities of VBM and CBM.

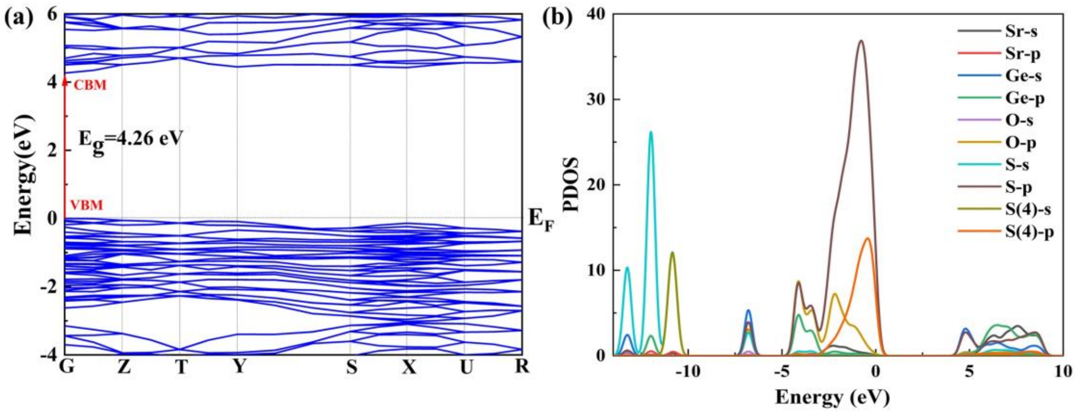

Figure S7. The theoretical calculations of $Sr_3S[GeOS_3]$ by HSE06 functional.

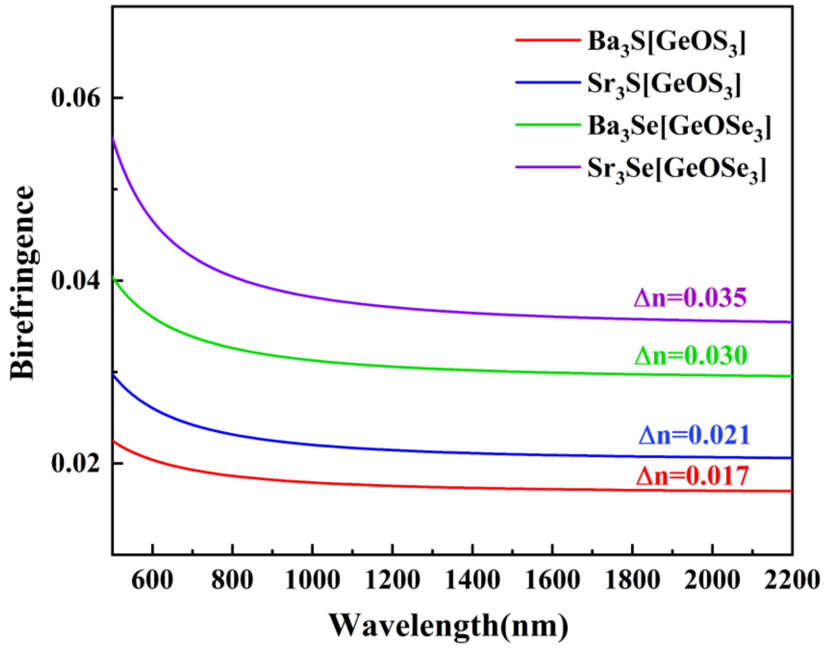

Figure S8. Calculated wavelength-dependent birefringence Δn for title compounds.

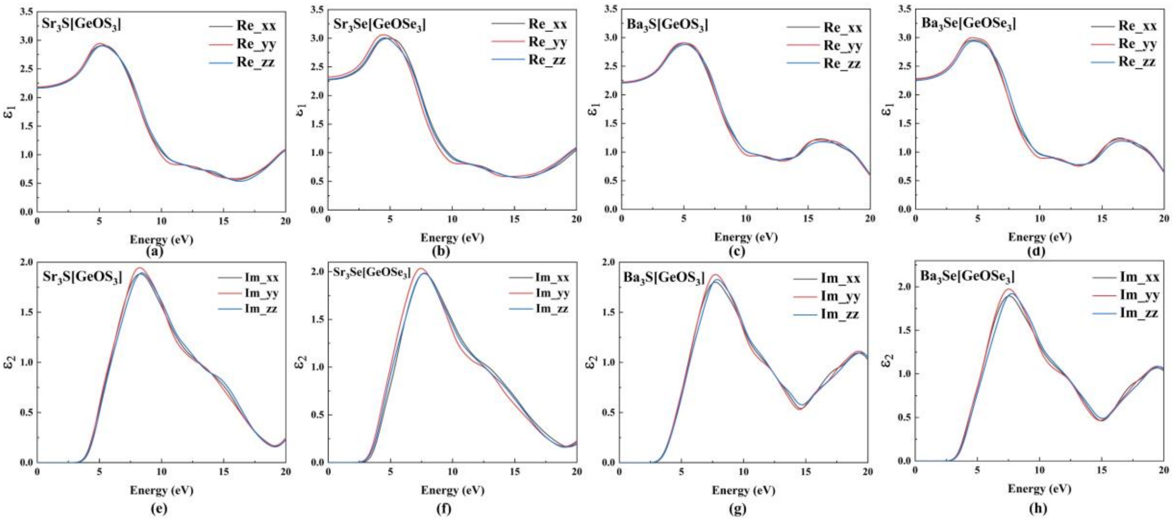

Figure S9. Energy dependences of the real part ε_1 (a-d) and the imaginary part ε_2 (e-h) of title compounds.

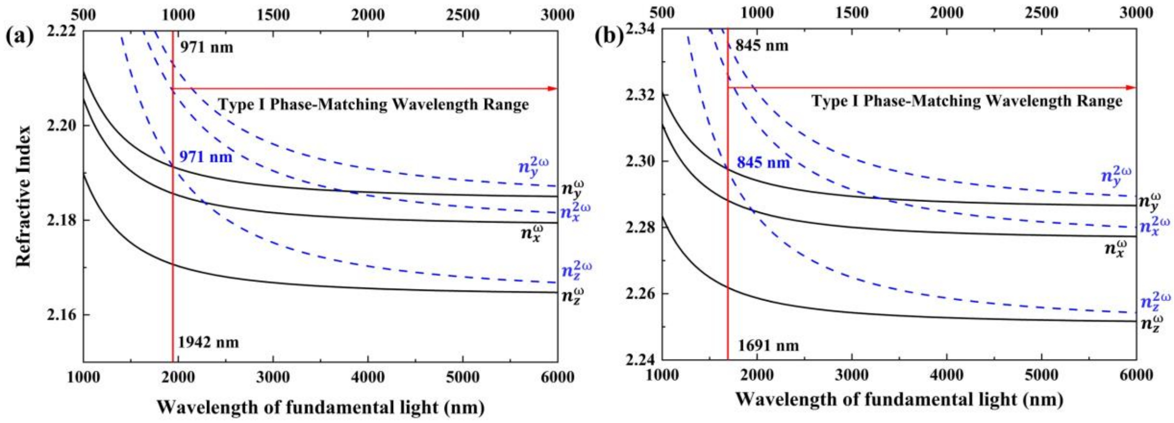

Figure S10. The calculated phase matching of (a) $Sr_3S[GeOS_3]$ and (b) $Sr_3Se[GeOSe_3]$ based on the first principles.

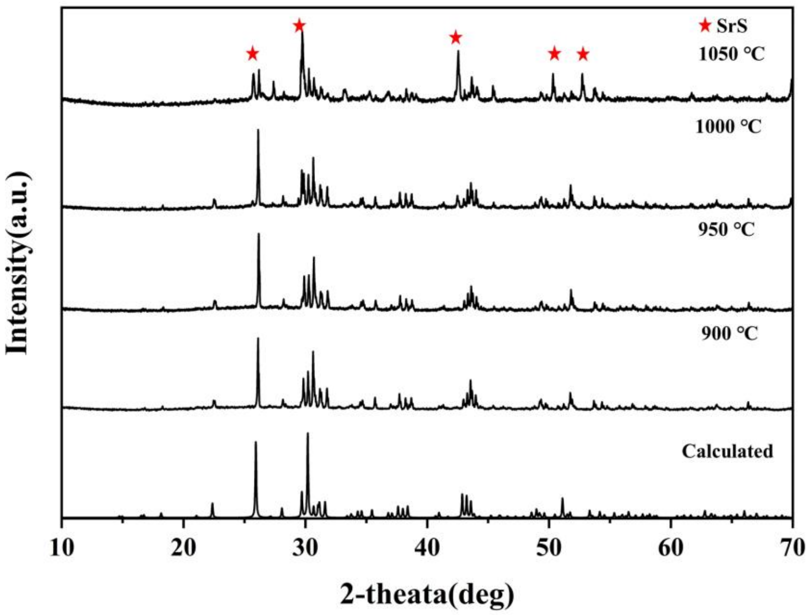

Figure S11a. The powder X-ray diffraction patterns of $Sr_3S[GeOS_3]$ at different temperature.

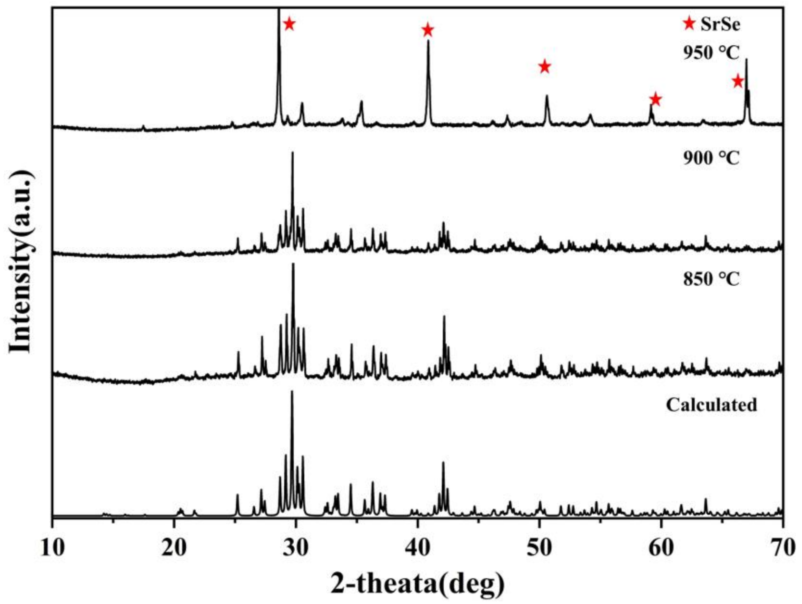

Figure S11b. The powder X-ray diffraction patterns of $Sr_3Se[GeOSe_3]$ at different temperature.

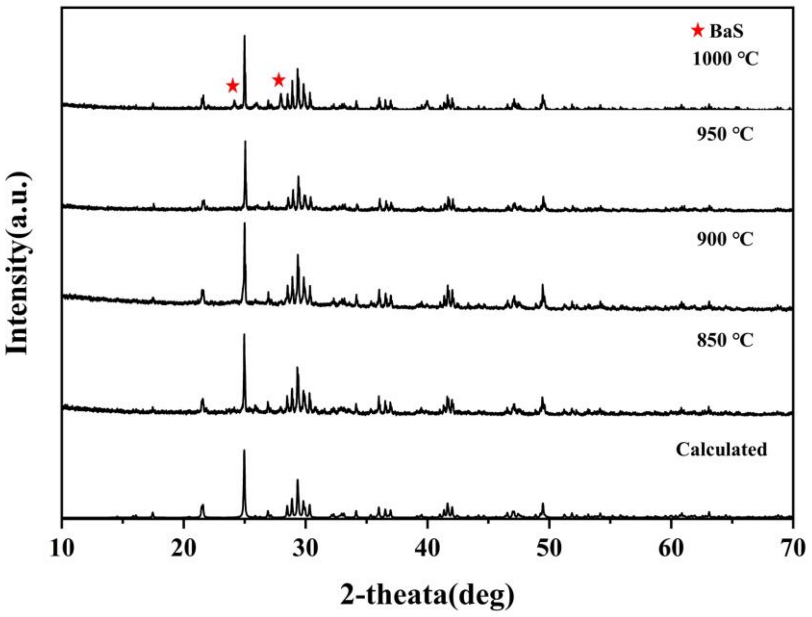

Figure S11c. The powder X-ray diffraction patterns of $Ba_3S[GeOS_3]$ at different temperature.

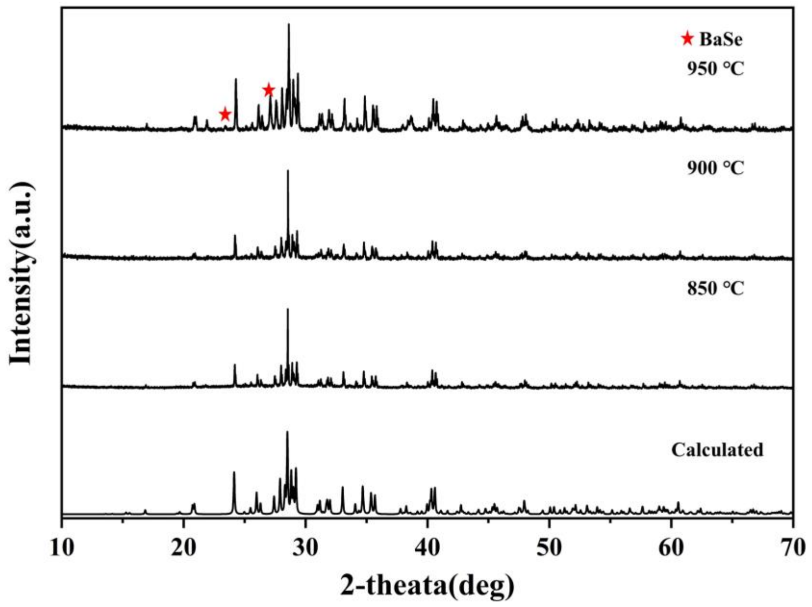

Figure S11d. The powder X-ray diffraction patterns of $Ba_3Se[GeOSe_3]$ at different temperature.

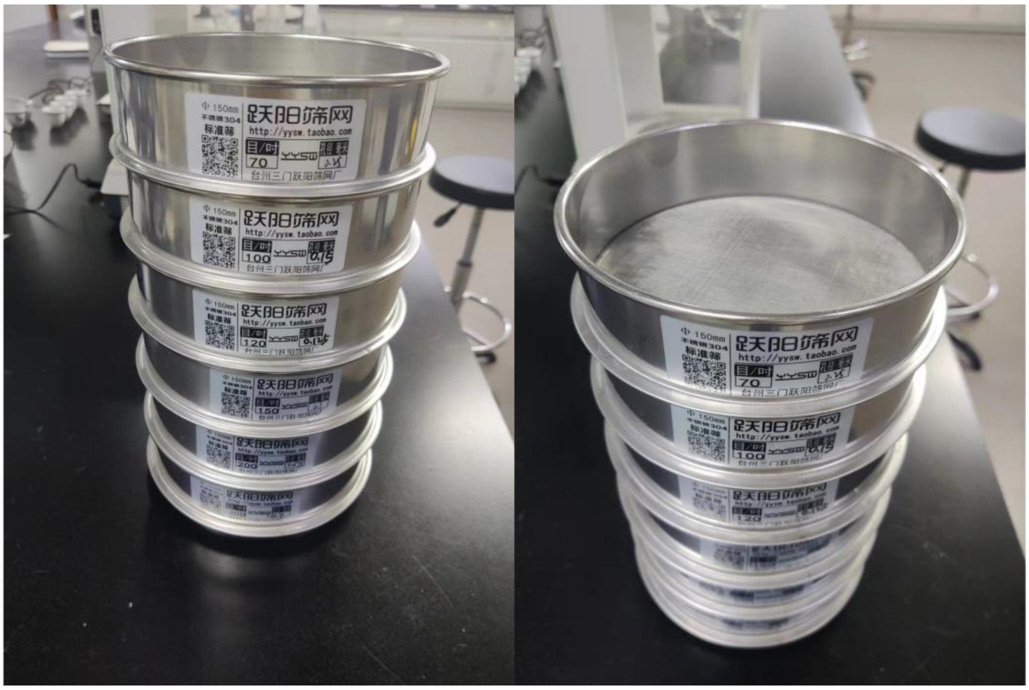

Figure S12. Used for screening different size sieve, the size ranges are 54-75 μm, 75-100 μm, 100-125 μm, 125-150 μm, and 150-250 μm, respectively.

Reference

[1] I. D. Brown, D. Altermatt, *Acta. Crystallogr. B* **2010**, *41*, 244.

[2] K. Wu, B. Zhang, Z. Yang, S. Pan, *J. Am. Chem. Soc.* **2017**, *139,* 14885

[3] J. Xu, K. Wu, Y. Xiao, B. Zhang, H. Yu, H. Zhang, *ACS Appl. Mater. Inter.* **2022**.

[4] X. Zhang, Y. Xiao, R. Wang, P. Fu, C. Zheng, F. Huang, *Dalton Trans.* **2019**, *48*, 14662.

[5] B. W. Liu, X. M. Jiang, G. E. Wang, H. Y. Zeng, M. J. Zhang, S. F. Li, W. H. Guo, G. C. Guo, *Chem. Mater.* **2015**, *27*, 8189.

[6] M. Y. Ran, Z. Ma, H. Chen, B. X. Li, X. T. Wu, H. Lin, Q. L. Zhu, *Chem. Mater.* **2020**, *32*, 5890.

[7] M. Y. Ran, S. H. Zhou, B. Li, W. Wei, X. T. Wu, H. Lin, Q. L. Zhu, *Chem. Mater.* **2022**, *34*, 3853.

[8] Y. Cheng, H. Wu, H. Yu, Z. Hu, J. Wang, Y. Wu, *Chem. Sci.* **2022**, *13*, 5305.

[9] W. Xing, P. Fang, N. Wang, Z. Li, Z. Lin, J. Yao, W. Yin, B. Kang, *Inorg. Chem.* **2020**, *59*, 16716.

[10] R. Wang, Y. Guo, X. Zhang, Y. Xiao, J. Yao, F. Huang, *Inorg. Chem.* **2020**, *59*, 9944.

[11] M. Sun, X. Zhang, C. Li, W. Liu, Z. Lin, J. Yao, *J. Mater. Chem. C* **2022**, *10*, 150.

[12] R. Wang, F. Liang, X. Liu, Y. Xiao, Q. Liu, X. Zhang, L. M. Wu, L. Chen, F. Huang, *ACS Appl. Mater. Inter.* **2022**.

[13] B. Almoussawi, W. D. Yao, S. P. Guo, M. H. Whangbo, V. Dupray, S. Clevers, S. Deng, H. Kabbour, *Chem. Mater.* **2022**, *34*, 4375.

[14] Y. Tsujimoto, C. A. Juillerat, W. Zhang, K. Fujii, M. Yashima, P. S. Halasyamani, H. C. zur Loye, *Chem. Mater.* **2018**, *30*, 6486.

[15] T. Sambrook, C. F. Smura, S. J. Clarke, *Inorg. Chem.* **2007**, 2571.

[16] J. Wang, Y. Cheng, H. Wu, Z. Hu, J. Wang, Y. Wu, H. Yu, *Angew. Chem. Int. Ed.* **2022**, *61*, e202201616.

[17] R. Wang, F. Liang, F. Wang, Y. Guo, X. Zhang, Y. Xiao, K. Bu, Z. Lin, J. Yao, T. Zhai, F. Huang, *Angew. Chem. Int. Ed.* **2019**, *58*, 8078.
